# Small molecule antagonists of PTPmu identified by artificial intelligence-based computational screening block glioma cell migration and growth

**DOI:** 10.1371/journal.pone.0288980

**Published:** 2023-07-26

**Authors:** Kathleen Molyneaux, Christian Laggner, Jason Vincent, Susann Brady-Kalnay

**Affiliations:** 1 Department of Molecular Biology & Microbiology, Case Western Reserve University, Cleveland, Ohio, United States of America; 2 Atomwise Inc., San Francisco, California, United States of America; UTHSC: The University of Texas Health Science Center at Houston, UNITED STATES

## Abstract

PTPmu (PTPμ) is a member of the receptor protein tyrosine phosphatase IIb family that participates in both homophilic cell-cell adhesion and signaling. PTPmu is proteolytically downregulated in glioblastoma generating extracellular and intracellular fragments that have oncogenic activity. The intracellular fragments, in particular, are known to accumulate in the cytoplasm and nucleus where they interact with inappropriate binding partners/substrates generating signals required for glioma cell migration and growth. Thus, interfering with these fragments is an attractive therapeutic strategy. To develop agents that target these fragments, we used the AI-based AtomNetⓇ model, a drug design and discovery tool, to virtually screen molecular libraries for compounds able to target a binding pocket bordered by the wedge domain, a known regulatory motif located within the juxtamembrane portion of the protein. Seventy-four high-scoring and chemically diverse virtual hits were then screened in multiple cell-based assays for effects on glioma cell motility (scratch assays) and growth in 3D culture (sphere assays), and PTPmu-dependent adhesion (Sf9 aggregation). We identified three inhibitors (247678835, 247682206, 247678791) that affected the motility of multiple glioma cell lines (LN229, U87MG, and Gli36delta5), the growth of LN229 and Gli36 spheres, and PTPmu-dependent Sf9 aggregation. Compound 247678791 was further shown to suppress PTPmu enzymatic activity in an *in vitro* phosphatase assay, and 247678835 was able to inhibit the growth of human glioma tumors in mice. We propose that these three compounds are PTPmu-targeting agents with therapeutic potential for treating glioblastoma.

## Introduction

Phosphorylation of specific amino acids (tyrosine, serine, threonine) in proteins is a well-known signal transduction mechanism for controlling protein function. This process, regulated by kinases and phosphatases, controls a wide-range of cell behaviors, including division and migration, that are important for development and normal physiology. However, disruption of kinase/phosphatase signaling cascades is a common feature in many disorders, including cancer. This has driven the development of therapeutic agents specifically designed to inhibit the catalytic activity of kinases [1], but attempts to target phosphatases, particularly tyrosine phosphatases, have lagged. The active sites of tyrosine phosphatases are both highly conserved and highly charged, meaning agents capable of targeting these sites in biochemical assays are often promiscuous and not suitable for in vivo use because they are unable to cross cell membranes [2, 3]. Thus, drug development in the phosphatase field has begun to focus on regulatory sites outside of the catalytic domain [4].

In this sense, the protein tyrosine phosphatase mu (PTPμ), a member of the IIb receptor protein tyrosine phosphatase (RPTP) family, is an attractive drug target. It has established structural motifs, defined by crystallography and deletion analysis, outside of its catalytic domain that could be exploited as targets, and it plays an important role in the development of several cancers [5–9] Structurally, PTPμ is member of a larger superfamily of RPTPs comprised of 21 genes in humans [10–13] subdivided into types (Type 1/VI, IIa, IIb, III, IV, V, VII and VIII) based on the sequences of their extracellular [14] and intracellular (http://ptp.cshl.edu/protein.shtml) domains. Members of the IIb subtype, including PTPμ, have structurally conserved extracellular domains (with only a moderate 49–63% a.a. similarity) [15] that contain an N-terminal meprin-A5-RPTPμ (MAM) domain followed by an Ig domain and four fibronectin type III repeats. The intracellular portion of PTPμ is comprised of two highly conserved phosphatase-like domains and a more divergent juxtamembrane region [16] (resembling the cytoplasmic region of cadherins [17]) predicted to have regulatory functions.

The extracellular domain of PTPμ mediates homophilic adhesion [16, 18, 19], with the MAM and Ig domains of one molecule interacting in trans with the first fibronectin repeat of another molecule [20]. The Ig domain mediates homophilic binding directly in vitro [19]. The MAM domain has been shown to mediate lateral (cis) interactions between PTPμ molecules within the same cell making an oligomeric functional adhesive complex [15, 21]. Engagement of adhesion via PTPμ is believed to be transmitted into changes in cell signaling via the catalytic activity of its membrane proximal phosphatase domain [22]. Its second phosphatase domain is thought to be catalytically inactive but may have regulatory or, as shown for RPTPT, alternative enzymatic (denitrase) [23] functions.

An additional regulatory structure, termed the wedge domain, is present within the juxtamembrane region of a subset of RPTPs (LAR, PTPμ, PTPα, PTPδ, PTPσ and CD45) [24], making it a more appealing target for specificity. The sequence of this region is more divergent than that of the tandem phosphatase domains [25] and it has predicted regulatory functions. Mutations in the wedge domain of CD45 prevented dimerization-induced inhibition of CD45 activity [26], and the crystal structure of the membrane proximal and D1 phosphatase domains of PTPα provides a structural rationale for this [27]. In this study, the wedge region was found to be inserted into the catalytic cleft likely blocking substrate access. However, more recent analysis of the structure of a CD45 construct (comprising the juxtamembrane, D1, and D2 domains) does not support this hypothesis [28]. Also, the crystal structures of the LAR juxtamembrane, D1, and D2 domains [25] and the PTPμ D1 region [29] did not reveal an inhibitory interaction between the wedge and catalytic cleft. For those RPTP constructs, the protein had an open conformation and was predicted to be catalytically active. The wedge domain has, however, been shown to participate in interactions between the D1 and D2 phosphatase domains [30], interactions that have been shown to be inhibitory for some RPTPs [31–33]. Finally, the wedge domains may control the interaction of RPTPs with other binding partners leading to changes in downstream signaling. For instance, a LAR-wedge domain peptide was able to block the interaction of LAR with TrkA leading to activation of tyrosine kinase dependent signaling in PC12 cells [24]. Likewise, a wedge peptide (Intracellular Sigma Peptide) directed against PTPσ was shown to affect signaling via Erks/CREB [34] and RhoA/CRMP2 [35] and is a promising agent for promoting neural regeneration after injury [36]. Thus, it is possible that the wedge domain of PTPμ could control interactions with its binding partners/substrates, which includes cadherins [17, 37], p120 catenin [38], PKCδ [39], PLCγ [39], IQGAP [40], and RACK1 [41], with therapeutic potential.

**Fig 1:**
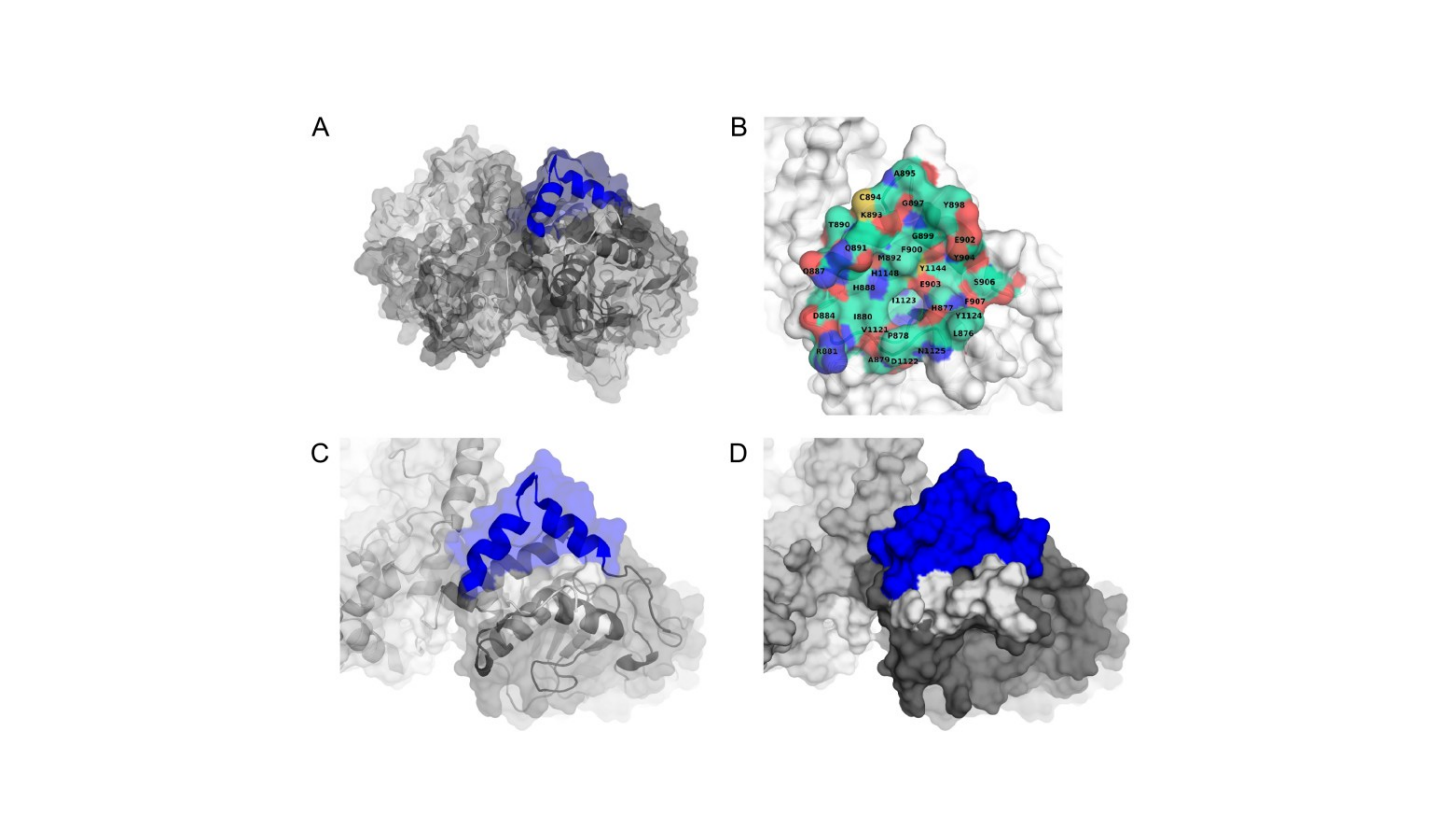
The PTPμ regulatory wedge domain borders a druggable pocket. A. Structure of the PTPμ wedge domain (blue) and the D1 domain and relative position to the D2 domain (modelled after the D1 PTPμ D1 and PTPσ D2 structures, PDB IDs 1RPM and 2FH7, respectively) [29, 54]. B. A space filling model of the residues surrounding the wedge-adjacent potential binding pocket. Y1224 is at the deepest position within the pocket. C. and D. The druggable cleft relative to the position of the wedge domain.

Targeting the wedge domain of PTPμ is an attractive strategy for treating malignancies. PTPμ expression is reduced in several forms of cancer (prostate [42], ovarian [43], endometrial [43], melanoma [44], and glioblastoma [45]). This suggests that PTPμ acts as a tumor suppressor, possibly by regulating adhesive interactions necessary for contact-dependent suppression of cell migration and/or growth [6, 8, 9]. In some cancers, however, the loss of PTPμ is proteolytic, and both extracellular and intracellular fragments of PTPμ are retained in tumors [46]. These fragments have been exploited to serve as prognostic biomarkers [47] and imaging agents [48–52], but they are not just inert proteolytic byproducts. An shRNA-mediated reduction of PTPμ in a glioma cell line (LN229) (that expresses mostly PTPμ fragments) was shown to reduce cell migration and growth factor independent growth [46], suggesting the fragments have oncogenic activity. A small peptide directed against the wedge domain of PTPμ was also able to block migration and growth-factor independent survival of LN229 cells, indicating aberrant signaling via an intracellular fragment, which can accumulate in the nucleus [46], may drive these processes by interacting with inappropriate substrates. Importantly, the PTPμ wedge peptide did not interact with the LAR wedge region [24], suggesting this domain could be a highly specific drug target. To exploit this, we used the AtomNet® platform [53], a deep learning artificial intelligence neural network for structural based drug design, to computationally screen for small molecules predicted to interact with a binding pocket bordered by the wedge domain of PTPμ (Fig 1) and tested these compounds in multiple cell-based assays. We identified three compounds (247678835, 247682206, 247678791) able to inhibit glioma cell migration, growth in non-adherent cultures, and, surprisingly, PTPμ-dependent adhesion. One of these compounds (247678791) was also found to modestly inhibit PTPμ’s catalytic activity in vitro, and one compound (247678835), the strongest identified in the screen, was found to inhibit glioma-cell growth in a human glioma tumor model in mice. We propose that these compounds represent specific PTPμ-targeting agents that can be further developed to treat cancers including glioblastoma.

## Results

### Identification of putative small molecule PTPμ inhibitors via AI-based virtual screening

A small pocket on the surface of PTPμ’s D1 domain (Fig 1), close to the wedge domain, was selected for virtual screening with the AtomNet® platform. This area sits at the interface between the D1 domain and the neighboring juxtamembrane domain for which no suitable modeling templates exist. Hence, only one half of what may be an inter-domain groove could be used for the virtual screening. The D2 domain and parts of the N-terminal linker domain were modeled after the crystal structure for the related PTPσ whereas the D1 domain was based on the available crystal structure for the PTPμ D1 domain (PDB IDs 2FH7 and 1RPM, respectively) [29]. ICM (v3.8–7 Molsoft L.L.C. San Diego, USA) was used for the homology modeling.

Atomwise used their proprietary AI screening AtomNet® platform to screen 4 million compounds from the Mcule small-molecule library (version v20171018, https://mcule.com/) as described previously [53, 55, 56]. The 2,000 top-scoring compounds were processed as follows: Compounds containing undesired (potentially reactive, unstable, or promiscuous) chemical moieties were removed. A pose filter was applied to select for compounds that are within a 4 Å heavy-atom distance from the H888 sidechain to select for those binding closely to the wedge domain and near the deepest indentation of the selected screening site. ECFP4 fingerprint-based Butina clustering using a Tanimoto coefficient of 0.4 for similarity cutoff was used to arrive at a final selection of 74 chemically diverse compounds [57]. The selected compounds were provided as 10 mM DMSO stocks together with 2 DMSO controls as blinded samples.

### Overview of the PTPμ-inhibitor screen

**Fig 2:**
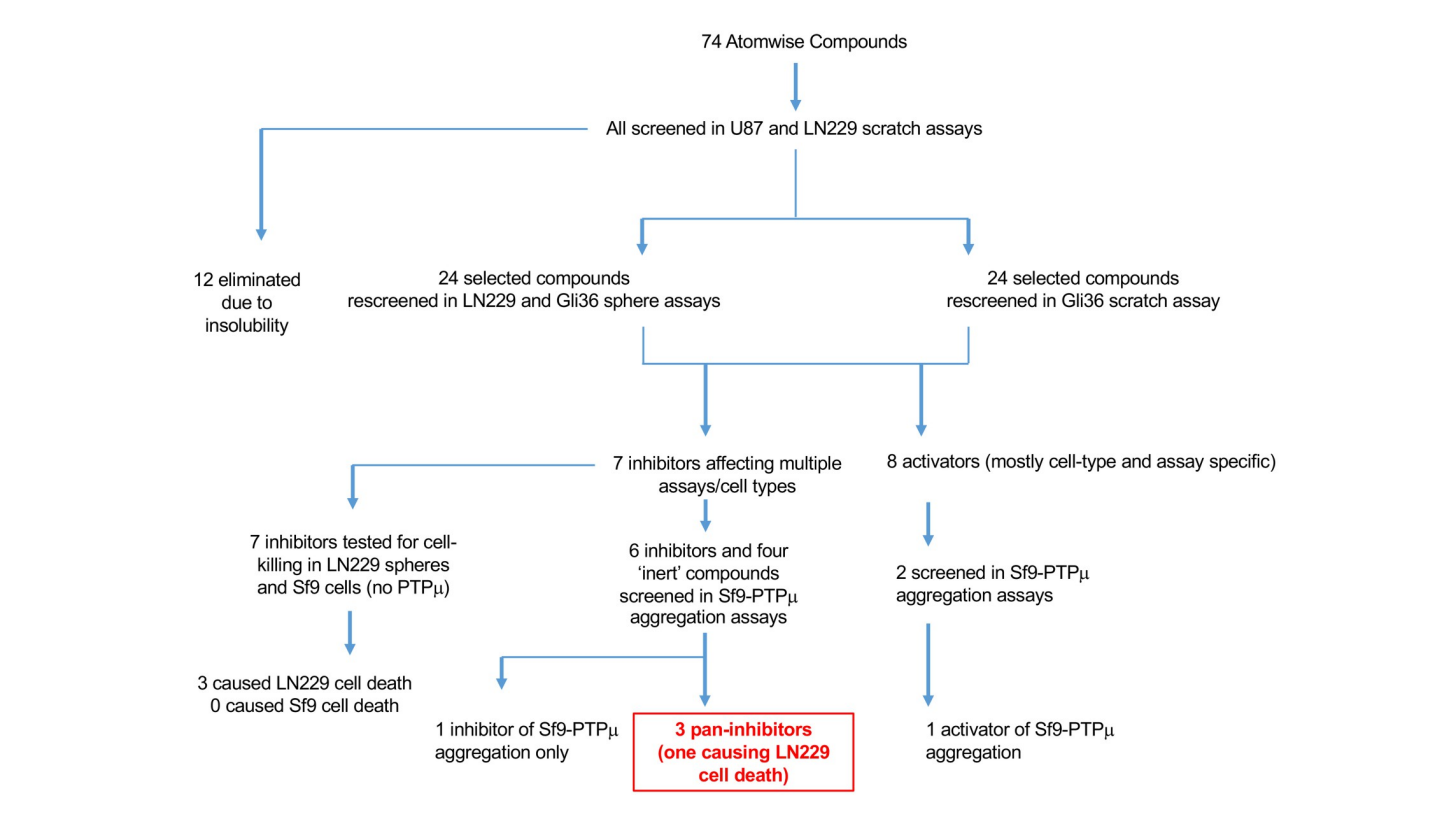
Functional screening approach. Seventy-four PTPμ wedge-targeted compounds and 2 blinded DMSO samples were received from Atomwise and screened at 100 μM in scratch wound healing assays using two glioma cell lines (LN229 and U87). Selected active and control compounds were taken into secondary assays (Gli36 scratch and LN229 and Gli36 sphere formation and growth assays). Inhibitors selected as being active in primary and secondary screens were tested for effects on the survival of LN229 and Sf9 cells, and selected glioma-cell inhibitors and activators were screened for effects on Sf9-PTPμ aggregation, a highly specific test for PTPμ function. These assays identified 3 high priority compounds (247678835, 247682206, 247678791) able to inhibit glioma cells and affect PTPμ-mediated aggregation. We also identified one compound able to inhibit PTPμ-mediated aggregation (247685429) that did not affect glioma cells and one compound (247685114) able to activate PTPμ-mediated aggregation and stimulate Gli36 sphere formation and growth.

**Fig 3:**
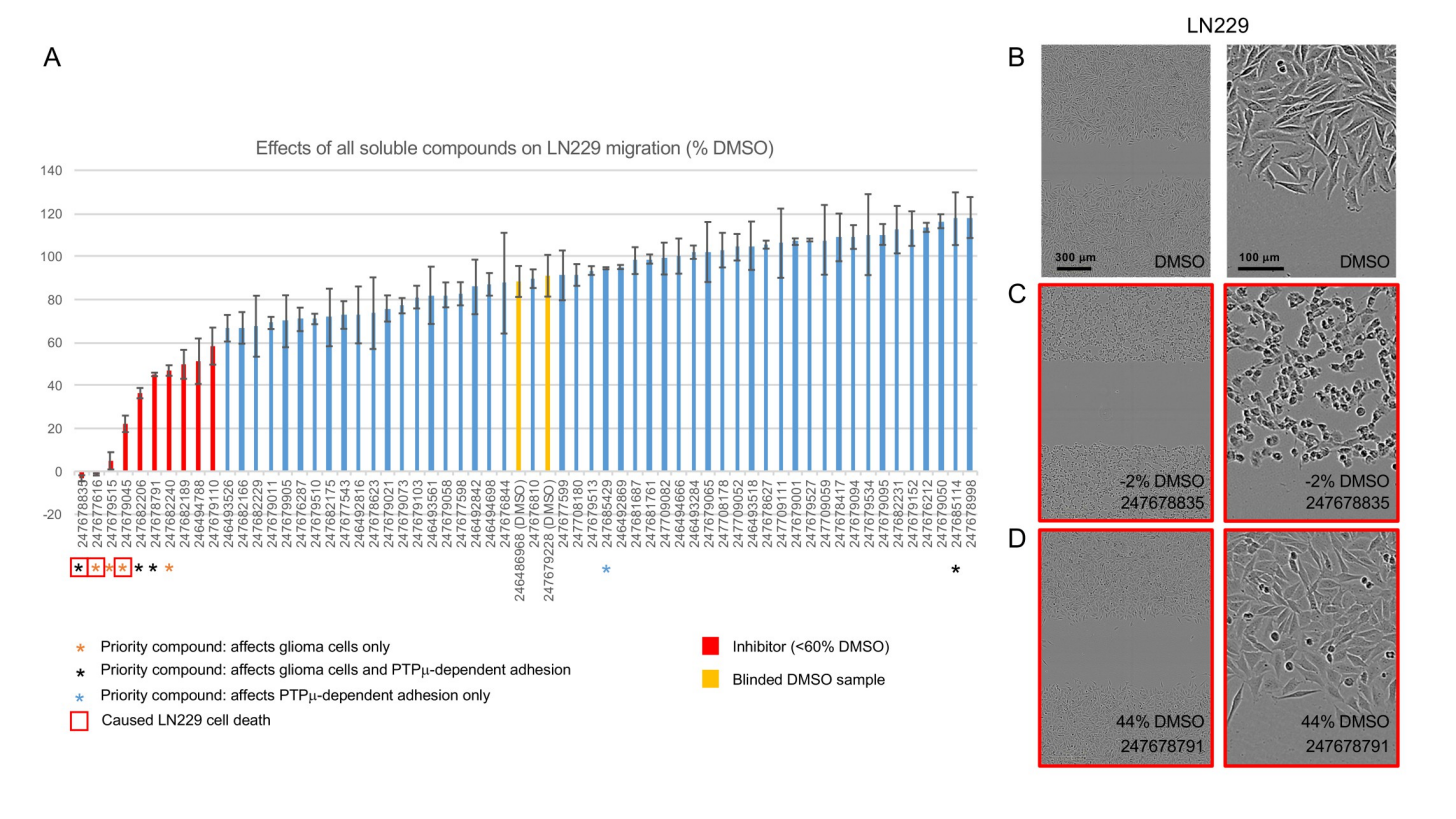
LN229 scratch wound assays. A. Histogram showing the effects of all soluble wedge-targeted compounds on LN229 scratch wound closure. Cell movement into the scratches was quantified from scratch wound widths at the start and end of the assay and normalized to the average movement of cells in the unblinded DMSO control samples. Data is presented as average percentages ± standard error of the means (s.e.m.), and compound bar codes are shown on the x-axis. The majority of compounds were screened with an n = 2. Some priority compounds were screened with 3–6 replicates. Representative endpoint images of samples treated with DMSO (B) and two priority inhibitors (C and D) are shown. The relative migration distances for each example are indicated.

Seventy-four compounds computationally predicted to interact with the PTPμ binding pocket near the wedge domain and 2-blinded DMSO controls were received from Atomwise and screened (at 100 μM) for activity in multiple cell-based assays (Fig 2). A non-blinded DMSO sample was used for normalization purposes in all experiments. In our primary screen, we tested the effects of the compounds on the migration of two different glioma cell lines LN229 (Fig 3 and S1 Fig) and U87MG (U87, S2 and S3 Figs) using a scratch wound healing assay. These cell lines were chosen because they express different levels of full-length PTPμ and its fragments and have different invasive behaviors in orthotopic tumor models. LN229 cells express mainly PTPμ fragments [45] and are invasive [58]; whereas, U87 cells express full-length and some PTPμ fragments [45] and exhibit little invasive behavior in vivo [58]. A peptide designed to target the wedge domain of PTPμ was shown to reduce LN229 migration in scratch assays by blocking the oncogenic activity of intracellular PTPμ fragments [46]; thus, we expected compounds able to bind the wedge pocket to have a similar effect.

**Fig 4:**
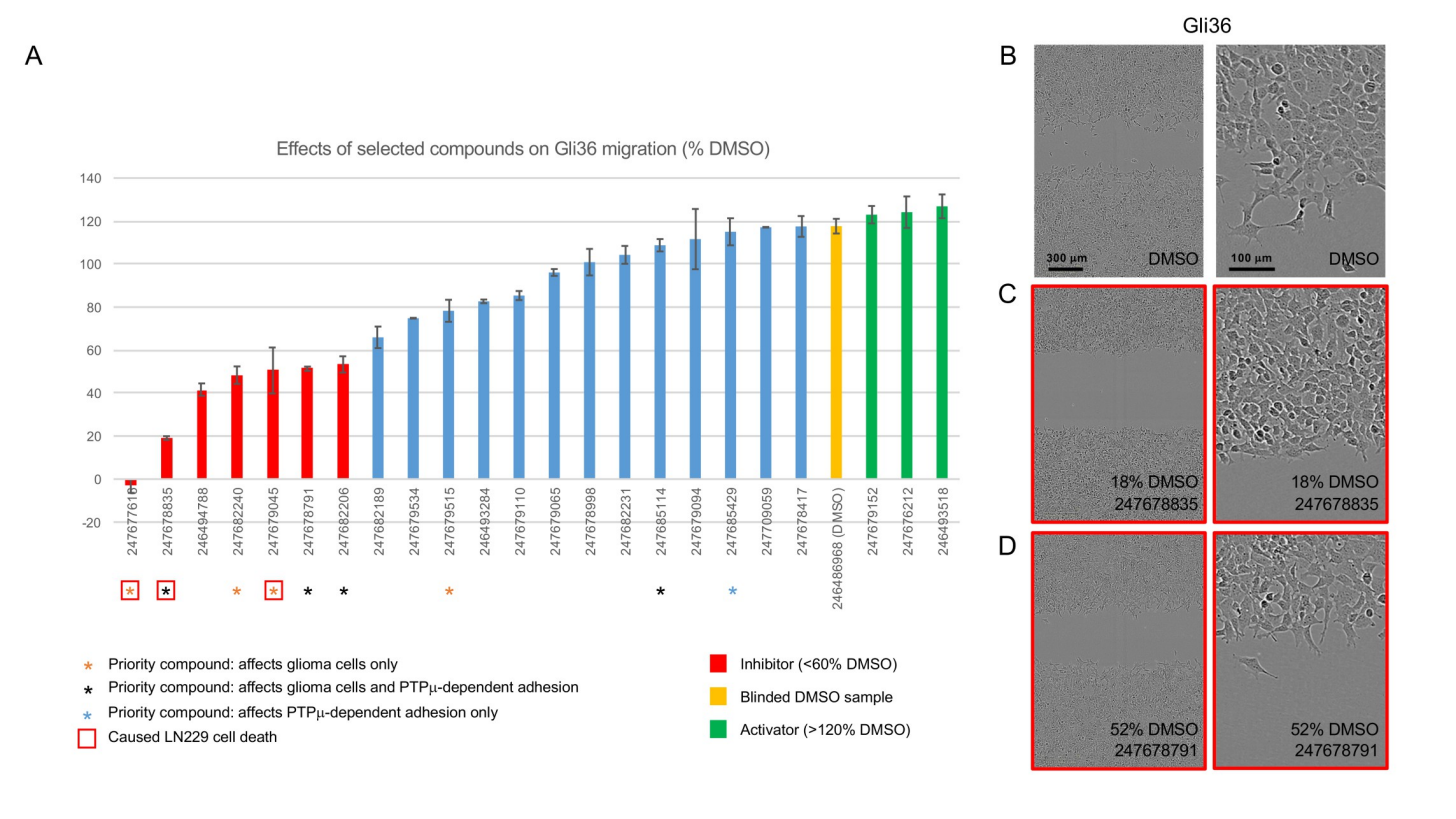
Gli36 scratch wound assays. Selected inhibitors, activators, and control compounds identified in LN229 and/or U87 scratch wound closure assays were retested at 100 μM for effects on Gli36 migration. A. Histogram showing the normalized migration distance for each treated sample. Data is presented as average % movement ± s.e.m, and compound bar codes are shown on the x-axis. The majority of compounds were screened with an n of 2–4. Representative endpoint images of samples treated with DMSO (B) and two priority inhibitors (C and D) are shown. The relative migration distances for each example are shown.

**Fig 5:**
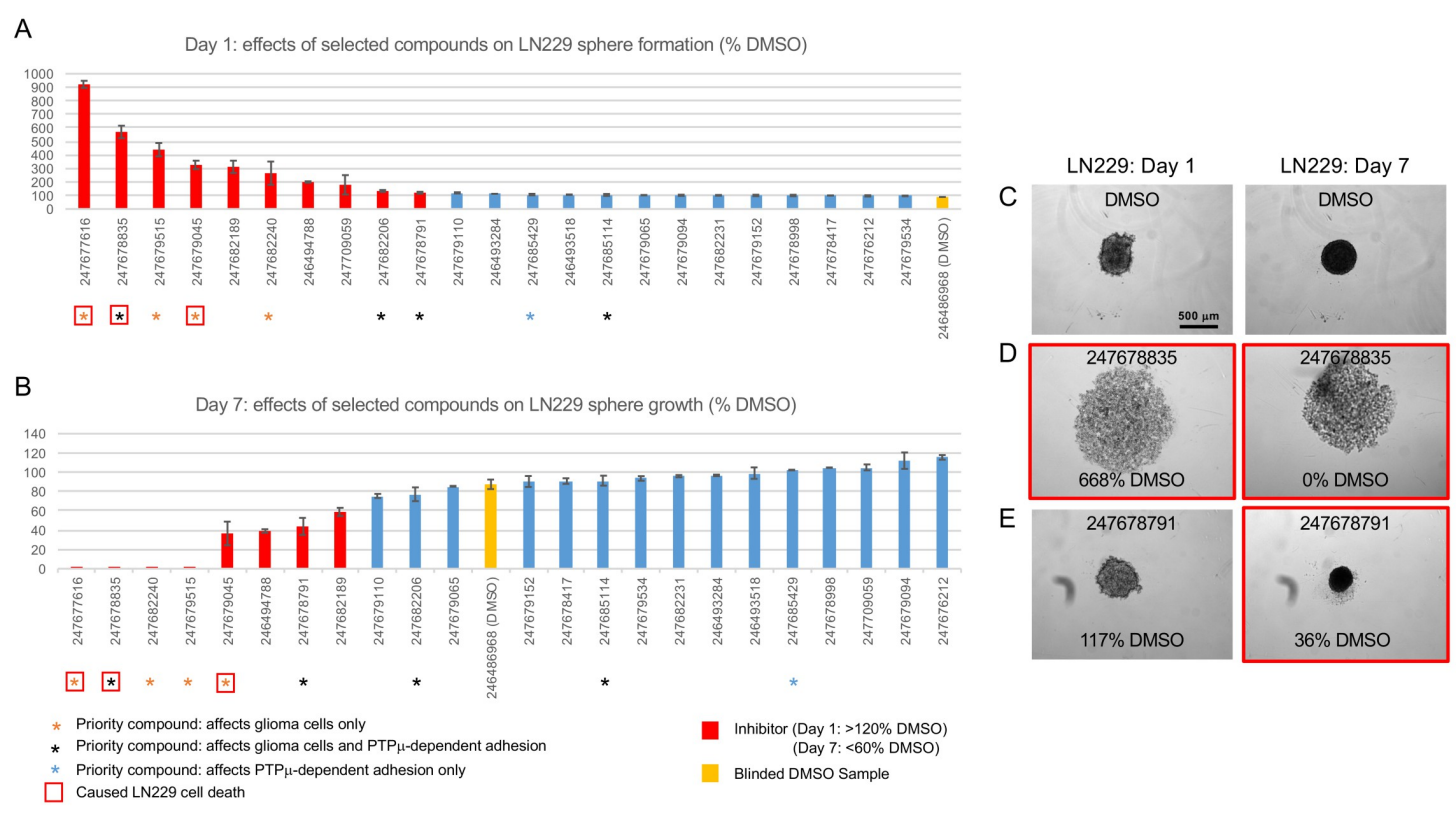
LN229 sphere formation and growth assays. Selected inhibitors, activators, and control compounds identified in LN229 and/or U87 scratch wound closure assays were retested at 100 μM in a secondary assay for glioma cell (LN229) sphere formation and growth. A. Histogram showing the effects of the indicated compounds on sphere formation. On day 1, sphere footprint areas were determined and normalized to the average footprint area of the unblinded vehicle-treated controls. On day 1, a larger footprint area indicates inhibition of aggregation. B. Histogram showing the effects of the indicated compounds on sphere growth. On day 7, the changes in sphere footprint areas were calculated and normalized to the average size change of the unblinded vehicle-treated controls. On day 7, a smaller value indicates reduced growth. Growth could not be calculated for samples that fell apart on day 1 or during the assay, and this is indicated as ‘0’ growth. Data is presented as percentages ± s.e.m. of 2–4 replicates. Representative images of samples treated with DMSO (C) and two priority inhibitors (D and E) are shown. Relative day 1 footprint areas and day 7 growth measurements are indicated for each example.

**Fig 6:**
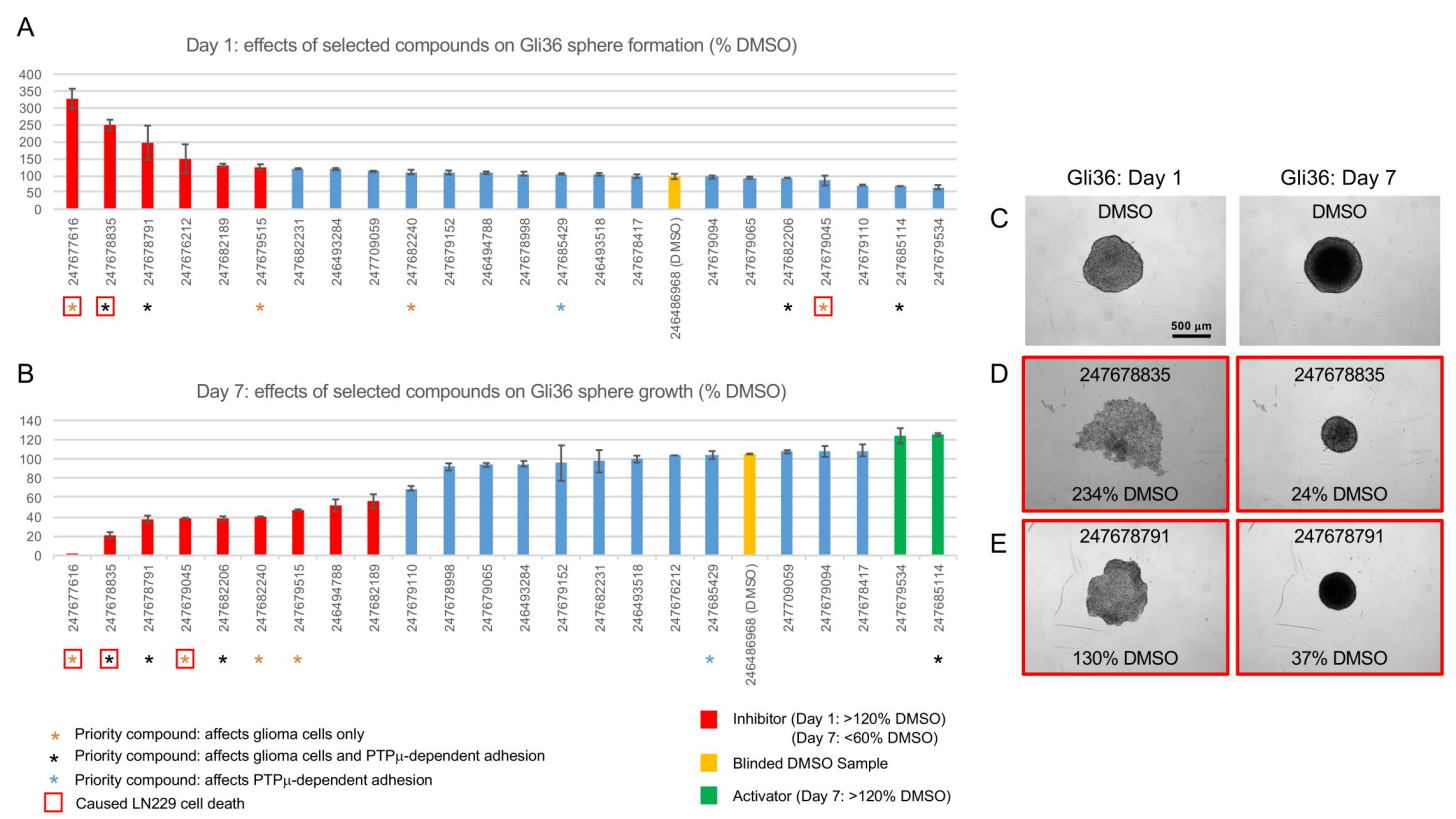
Gli36 sphere formation and growth assays. Selected inhibitors, activators, and control compounds identified in LN229 and/or U87 scratch wound closure assays were retested at 100 μM in a secondary assay for glioma cell (Gli36) sphere formation and growth. A. Histogram showing the effects of the indicated compounds on Day 1 sphere footprint areas. Footprint areas were measured and normalized to the average footprint area of the unblinded DMSO-treated controls. On day 1, a larger footprint area indicates inhibition of aggregation. B. Histogram showing the effects of the indicated compounds on sphere growth. Changes in sphere footprint areas were calculated and normalized to the average size change of the unblinded vehicle-treated controls. On day 7, a smaller value indicates reduced growth. For the samples that fell apart on day 1 (247677616), growth could not be calculated and this is indicated as ‘0’ growth. Data is presented as percentages ± s.e.m. of 2–4 replicates. Representative images of samples treated with DMSO (C) and two priority inhibitors (D and E) are shown. Relative day 1 footprint areas and day 7 growth measurements are indicated for each example.

Of the 74 compounds, twelve were eliminated from the screen due to insolubility (S4 Fig), and 24 compounds (11 inhibitors of one/both cell types, four activators (all from the U87 motility screen), and nine compounds that had no effect to serve as controls) were selected for further testing on an additional cell line Gli36δ5 (Gli36) (Fig 4 and S5 Fig) and in an additional assay, glioma cell sphere formation and growth (Figs 5 and 6, S6 and S7 Figs). Like LN229 cells, Gli36 cells have very little full-length PTPμ but express fragments [48] and we expected the sensitivity profile of these cells to be similar to that of the LN229 cells. The glioma cell sphere formation and growth assay was selected as a secondary screening modality as it tests cell-cell adhesion and the ability to grow in 3-dimensions, creating a structure that more closely mimics a tumor and its microenvironment [59]. Compounds that are active in this assay are more likely to be effective in vivo.

From the primary and secondary screens, we selected seven highly penetrant inhibitors (affecting primary and secondary assays and multiple cell types) (247678835, 247677616, 247679515, 247679045, 247682206, 247678791, and 247682240). We also identified nine, mostly weak, activators of which one (247679152) affected both Gli36 and U87 migration and one (247679534) affected U87 migration and Gli36 spheres. The remaining activators were cell type/assay specific: four specifically affected U87 cell migration (246493284, 247679103, 247708178, and 247679095), two specifically affected Gli36 migration (247676212, 246493518), and one affected Gli36 spheres (247685114). Compound 247685114 was later shown to activate Sf9-PTPμ aggregation and is thus likely to be PTPμ-specific making it a high priority compound. The relevance of the other activators is unclear, and most were not considered further because they are unlikely to have therapeutic potential.

The seven penetrant inhibitors were tested to see if they affected the survival of LN229 spheres and parental Sf9 cells (which lack PTPμ) (S8 Fig). Three inhibitors (247678835, 247677616, and 247679045) caused a qualitative increase in LN229-cell death but did not affect parental Sf9 cells. This screen rules out non-specific effects on cells that do not express PTPμ. The effect on LN229 cells suggests they had a PTPμ-dependent survival effect as changes in PTPμ expression have been shown to affect cell viability [60].

**Fig 7:**
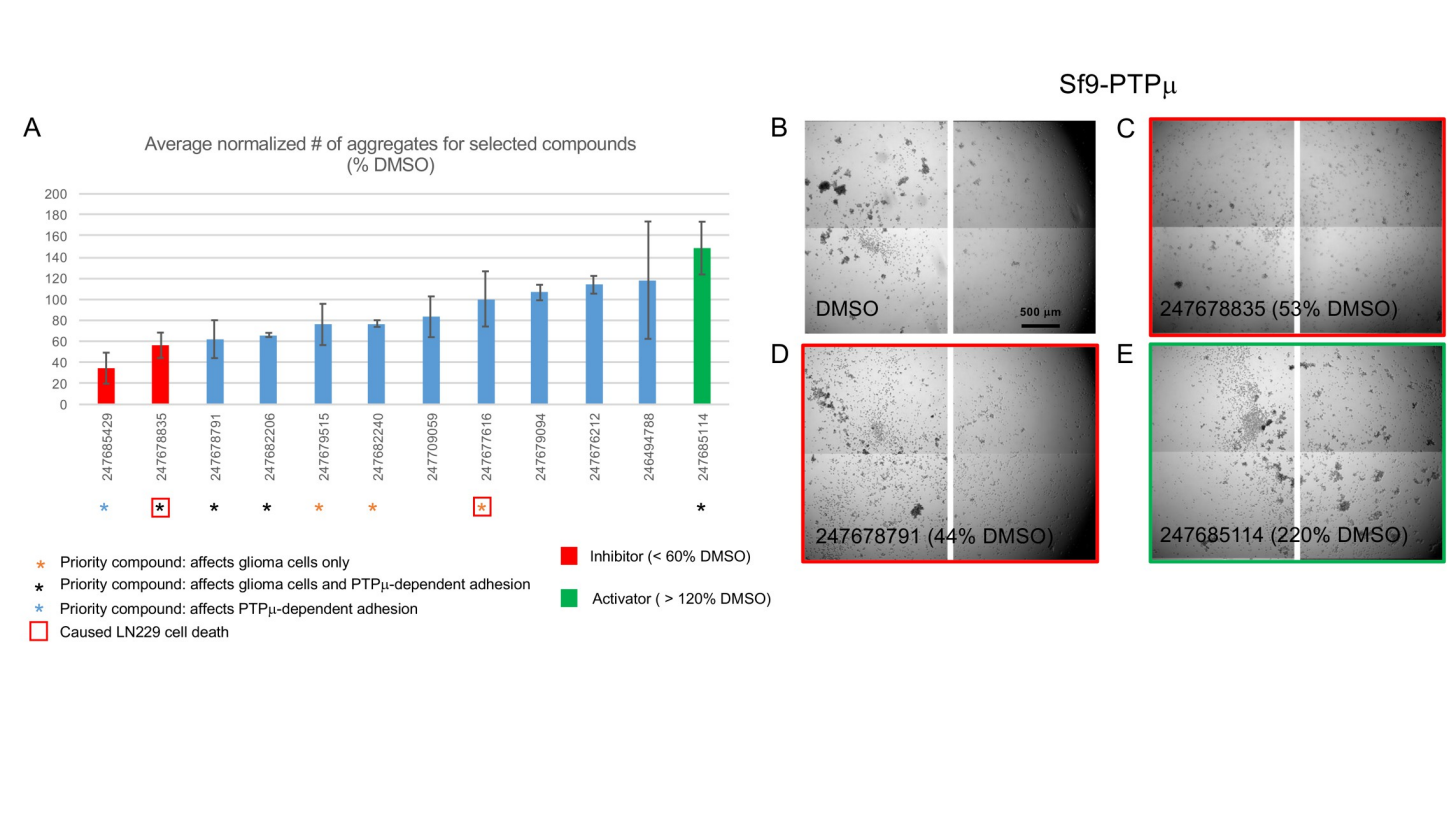
Testing the effects of selected compounds on PTPμ-dependent adhesion. Sf9 cells (which lack endogenous PTPμ) were infected with a baculovirus expressing full-length human PTPμ. Cells were harvested 48 h after infection, treated for 20 min with compounds (at 100 μM) or DMSO, and induced to aggregate by rotation. Wells were imaged as a 4x4 grid to capture the entire surface area. A. Histogram showing the effects of the selected compounds on PTPμ-dependent aggregation. Aggregates above an arbitrary footprint size (4000 μm^2^) were counted and normalized to the average number present in the DMSO-treated controls. Data is presented as percentages ± s.e.m. of 2–6 replicates. Representative images (central frames) of samples treated with DMSO (B), two priority inhibitors (C and D), and one priority activator (E) are shown. The relative number of aggregates for each example are shown.

To directly test PTPμ targeting, six of the penetrant inhibitors (black and orange asterisks Fig 7) and two activators (the Gli36 sphere-specific activator 247685114 and the strongest U87 migration-specific activator 247679095) were screened in a tertiary assay to test if the compounds can perturb PTPμ-mediated aggregation of Sf9 cells that are infected with a recombinant baculovirus to express PTPμ (Sf9-PTPμ Fig 7). Parental Sf9 cells lack PTPμ (as well as other RPTPIIb family members), thus this assay is highly specific. The wedge domain could regulate the enzymatic activity or intracellular binding partners of PTPμ but it is unclear how this might affect PTPμ’s adhesive function. Of the six tested penetrant inhibitors, only one met our strict cut-off for inhibition of Sf9-PTPμ aggregation (< 60% of the average DMSO control number of aggregates). However, two additional compounds nearly reached this threshold and are also considered high priority. We also identified one inhibitor of Sf9-PTPμ aggregation that did not affect glioma cells and, curiously, one activator of PTPμ-mediated aggregation. Compound 247685114 was identified as an activator of Gli36 sphere growth and moderately stimulated LN229 (Fig 3) and U87 cell migration (S2 Fig) (although it did not reach our strict cut-off for an activator (>120% DMSO) of cell motility).

Although, we had no expectation of identifying compounds able to perturb PTPμ’s adhesive function, we regard the compounds [3 inhibitors (247678835, 247682208, and 247678791) and 1 activator (247685114)] in this category as our highest priority hits because the Sf9 assay is a short-term assay that directly tests a known function of PTPμ and is less likely to be subject to any off-target effects. To aid tracking through the various assays, these high priority compounds are marked by black asterisks throughout the figures (Figs 3–7, S2 and S6 Figs). The four penetrant glioma cell inhibitors not shown to affect PTPμ-mediated aggregation (247677616, 247679045, 247682240, and 247679515) may still have therapeutic potential and are marked by orange asterisks. The compound that inhibited PTPμ-mediated aggregation but had no effect in glioma cell assays is indicated by a blue asterisk.

### LN229, U87, and Gli36 scratch assays

**Fig 8:**
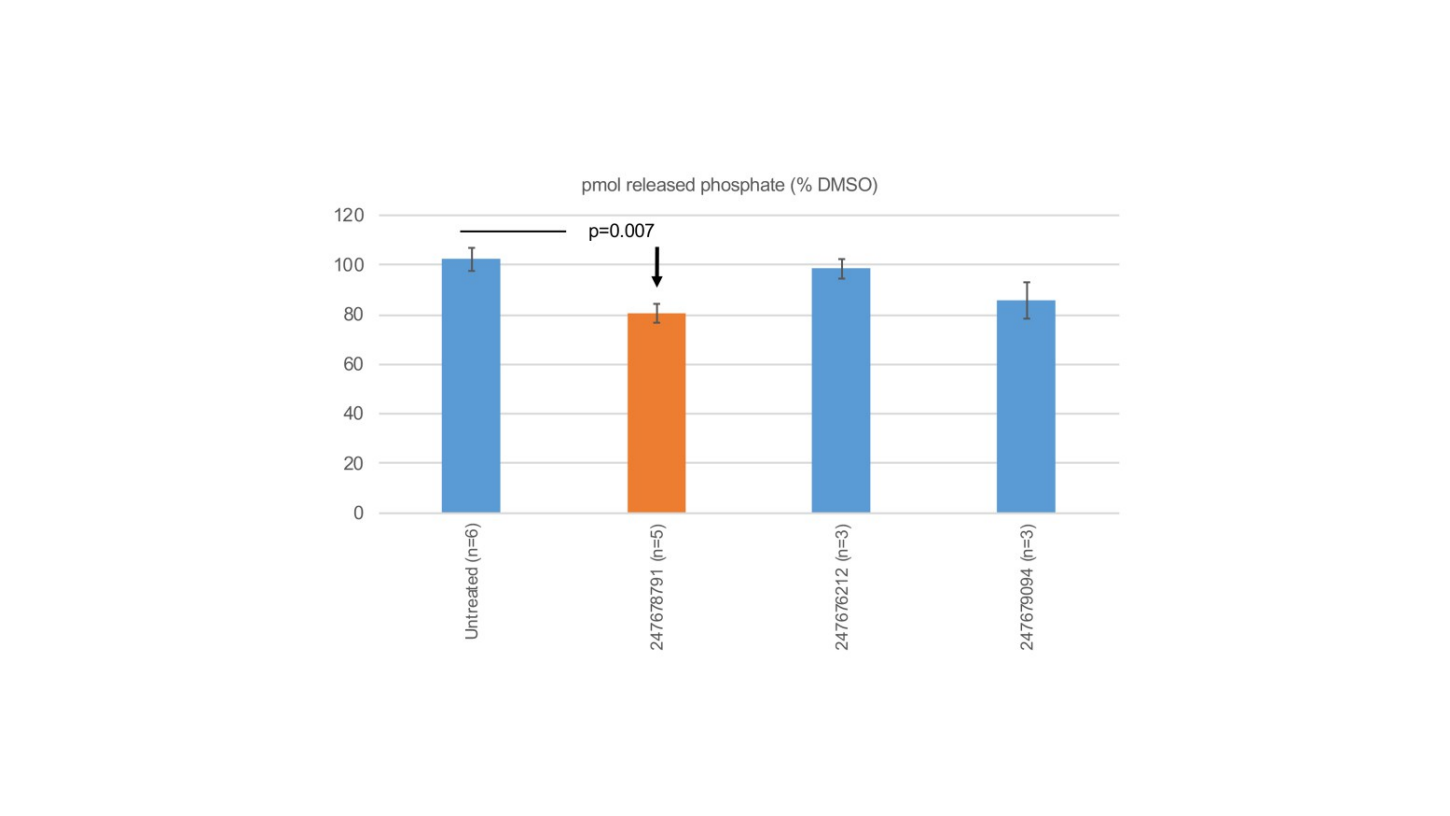
Phosphatase assays. Selected compounds were tested for their ability to affect the phosphatase activity of a GST-tagged PTPμ construct comprising the entire intracellular domain of human PTPμ. The enzyme was pretreated on ice for 10 min, and the reactions started by addition of a peptide substrate and incubation at 30° C. The amount of released phosphate was measured at 15 min using the malachite green reaction and normalized to that of the vehicle-treated control. Data is presented as percentages ± s.e.m. of the indicated number of independent experiments. Differences were assessed using the Student’s t-test with comparison to an untreated control sample (as all DMSO samples were set to 100% release). A difference was deemed significant at p<0.05. One priority compound (247678791) caused a modest, but statistically significant, reduction on enzymatic activity.

Scratch assays measure the ability of cells to migrate into a wound and close it creating a monolayer. Fig 3A shows the effects of all soluble wedge-targeting compounds on LN229 scratch wound closure, with the priority compounds indicated by asterisks as discussed above. In this initial screen, we identified 10 strong LN229 inhibitors that slowed wound closure to < 60% of controls (red bars) and, of these, 7 (247678835, 247677616, 247679515, 247679045, 247682206, 247678791, and 247682240) were eventually prioritized for being effective in multiple assays and on multiple cell types (Fig 2). Also, through the screening process, we identified one priority activator (247685114) based on its ability to activate PTPμ-dependent adhesion (Fig 8). This compound caused LN229 scratch wounds to close marginally faster (~120% of DMSO treated wounds).

There was considerable overlap between inhibitors able to affect LN229 and U87 cells (S2 Fig). However, the U87 cells seemed more ‘activatable’ than LN229s, with six compounds increasing U87 wound close rates by 20–50%. U87 cells do not form uniform monolayers, but instead grow to confluence as networks of cells connected by processes. These monolayers do not always wound cleanly and this likely accounts for the greater internal variability of the compound replicates using this cell type (see error bars in S2A Fig). Samples with poor replicates (s.e.m. > 10%) (which includes several compounds that seemed to be modestly (> 120%) or strongly (> 140%) activating) were not pursued further.

Representative end-point images of LN229 scratch wounds treated with DMSO (1%) or two high priority inhibitors (100 μM) are shown in Fig 3B–3D. Compound 247678835 (which was later found to affect LN229 survival, S8 Fig) was the strongest inhibitor of LN229 wound closure and completely blocked the movement of cells into the scratch. This was accompanied by obvious morphological changes. DMSO control-treated LN229 cells were generally spindle shaped within the monolayer, but those at the wound edge had a more flattened morphology and lamellopodial ruffles consistent with being migratory (Fig 3B). In contrast, cells treated with 247678835 were rounded with no ruffles (Fig 3C). Compound 247678791 (which did not affect LN229 survival, S8 Fig) had subtle effects on the morphology of LN229 cells: cells at the wound edge were more spindle-shaped than flattened and exhibited fewer ruffles (Fig 3D). Similar morphological changes were observed in U87 cells with these two priority compounds (S2C and S2D Fig).

The morphological effects of the other prioritized inhibitors on LN229 and U87 cells are shown in S1 and S3 Figs, respectively, and ranged from rounding [247677616 (S1B Fig) and 247679515 (S1F Fig)] to qualitatively fewer lamellipodia. Of note, some priority inhibitors [247682206 (S3D Fig) and 247682240 (S3E Fig)] seemed to cause a pile-up of U87 cells at the edge of the scratch wound (visible as what appears to be a chain of cells running parallel to the scratch). The priority activator did not produce obvious morphological changes in either LN229 or U87 cells. However, in the presence of this activator there did appear to be more individual U87 cells scattered within the scratch wound (S3G Fig) consistent with the modest average increase (~20%) in the rate of U87 wound closure seen with this compound.

All inhibitors identified in the primary screens, a selection of U87-specific activators, and some apparently inert control compounds were rescreened in scratch wound assays using an additional glioma cell line (Gli36) (Fig 4A). We found that the Gli36 cells migrated more rapidly than LN229 and U87 cells in scratch wound assays (requiring end point images to be taken at 8 h vs. the typical 12 h timeframe of the U87 and LN229 experiments). Despite this, Gli36 cells were still sensitive to the majority of priority inhibitors, but the priority activator did not affect these cells in this assay. Three weak Gli36 activators were identified, but only one (247679152) overlapped with those previously identified in U87 cells.

Fig 4B–4D shows representative images of Gli36 scratch wounds treated with DMSO and two selected priority inhibitors. In vehicle-treated control samples, the Gli36 cell monolayers had a cobblestone appearance, with cells at the scratch edge extending processes and appearing to move into the scratch as interconnected chains (Fig 4B). The strong priority inhibitor 247678835 reduced the appearance of processes and cell chains at the scratch edge (Fig 4C), while the moderate inhibitor 247678791 did not dramatically affect Gli36 cell morphology; processes were still present and short chains of cells were seen extending into the scratch (Fig 4D). The morphological effects of the other prioritized compounds are shown in S5 Fig. Of these, only 247677616 had a dramatic effect on Gli36 cell morphology. As seen in LN229 cells, this compound caused rounding and the appearance of intracellular phase dark areas (possibly indicating condensation/ fragmentation of nuclei) (S5B Fig). This compound was flagged as causing LN229 cell death (S8 Fig).

### LN229 and Gli36 sphere assays

We tested the effects of twenty-four compounds (11 flagged as inhibitory, 4 as stimulatory, and nine as inert in the initial scratch-wound screen, Fig 2) on the ability of LN229 cells to mediate cell-cell adhesion and grow in 3D culture on non-adherent surfaces. To quantify sphere formation (Fig 5A), the footprint areas of aggregates were measured on day 1 and normalized to that of the DMSO control. At this time point, a larger footprint size (>120%) indicates inhibition, i.e., the failure of cells to form a compact aggregate. To quantify sphere growth (Fig 5B), we calculated the percent changes in sphere footprint areas between day 1 and day 7 and normalized them to that of the DMSO controls. At this time point, compounds that reduced growth by >40% were considered inhibitory; however, growth could not be measured for samples that fell apart on day 1 or during the culture period. Compounds that caused either effect are displayed as having 0% growth in the graph.

Fig 5 shows representative images of LN229-cell aggregates cultured in the presence of DMSO (Fig 5C) or two selected priority inhibitors (Fig 5D and 5E). After one day in culture, the cells treated with DMSO had formed a loose aggregate, which by day 7 had grown into a compact sphere. In contrast, the cells treated with a strong inhibitor (247678835) failed to compact and formed a mat of cells at the bottom of the well. Samples treated with a moderate inhibitor (247678791) aggregated more slowly than controls (based on the modest relative increase in footprint size measured on day 1) and grew poorly in 3D culture. These two inhibitors were tested at different dosages (25 μM, 50 μM and 100 μM) to determine the minimal dose able to affect sphere formation (S6A and S6C Fig) and/or growth (S6B and S6D Fig). 247678835 dramatically disrupted sphere formation at 100 μM. This effect was still apparent at 50 μM but was less dramatic. At this dose, condensation was slowed and the resulting aggregates grew poorly in culture. 247678791 slowed aggregation at 100 and 50 μM but only reached our threshold for inhibition (< 60%) of sphere growth at 100 μM.

Representative images of samples treated with the other priority inhibitors (only tested at 100 μM) are shown in S7 Fig. These either completely blocked sphere formation (247677616, 247679515, 247679045) resulting in loose cells on day 1 or delayed sphere formation and inhibited growth (247682206 and 247682240), as evidenced by modestly larger footprint areas on day 1 but smaller spheres and/or loose cells on day 7. The priority activator 247685114 did not affect LN229 sphere formation or growth.

The priority inhibitors generally caused similar effects on Gli36 sphere formation and growth (i.e., slowed aggregation resulting in larger aggregates on day 1 and slowed sphere growth resulting in smaller aggregates on day 7) (Fig 6 and S7 Fig). The strong priority compound 247678835 slowed aggregation of Gli36 cells (Fig 6A and 6D); however, unlike LN229 cells treated with this compound, 247678835-treated Gli36 cells still eventually formed spheres. These spheres grew poorly (Fig 6B) and appeared more optically translucent than control spheres on Day 7 (Fig 6D). The moderate inhibitor 247678791 also slowed condensation of Gli36 cells (Fig 6A and 6E) and produced spheres that grew more slowly than controls (Fig 6B and 6E). The effects of the other priority compounds are shown in S7 Fig. Notably, the only compound that completely blocked Gli36 sphere formation was 247677616. The priority activator (247685114) seemed to accelerate Gli36 sphere condensation and growth. The average day 1 sphere footprint area of cells treated with this compound was 68% of the control area, and these spheres grew marginally faster than controls.

### PTPμ-dependent aggregation assays

Long-term cell-based assays are complex and can yield off-target effects/toxicity. We tested the effects of selected priority compounds in a short-term assay of PTPμ-dependent adhesion. Sf9 cells lack RPTPIIb family members and are not normally self-adherent but can be induced to aggregate by expressing PTPμ [16, 55]. This provides a highly-specific measure of PTPμ function. If the compounds had any effect on the dimerization, cis interactions, or cytoskeletal association of PTPμ they could impact PTPμ-dependent aggregation. Sf9 cells expressing PTPμ were treated with selected priority compounds (100 μM) for 20 min then induced to aggregate by rotation. The number of aggregates above an arbitrary threshold size (4000 μm^2^) were counted and normalized to the number present in the vehicle-treated controls (Fig 7A). Fig 7 shows representative endpoint images of samples treated with DMSO, 2 priority inhibitors, and one priority activator. In the DMSO-treated sample many variable-sized aggregates have formed (Fig 7B), but samples treated with the glioma-cell inhibitors 247678835 (Fig 7C) and 247678791 (Fig 7D) exhibit fewer/smaller aggregates. 247685114 was flagged as a modest activator of Gli36 sphere growth (Fig 6), but had only weak, if any, activity in other glioma-cell assays. Surprisingly, samples treated with this compound showed a considerable increase in aggregate numbers (Fig 7E), indicating that it can stimulate PTPμ’s adhesive function.

### PTPμ enzymatic activity

To test whether our priority compounds alter PTPμ’s enzymatic activity, we used an in vitro phosphatase assay (Fig 8). A GST-tagged protein corresponding to the entire intracellular domain of human PTPμ was preincubated on ice with DMSO or selected compounds (100 μM) and then the reaction started by addition of a peptide substrate and incubation at 30° C. At the endpoint of the assay, released phosphate was measured using a colorimetric reaction (the malachite green assay) and normalized to the amount released by the vehicle-treated control. The data shown represents the results of 3–6 independent experiments. One of our high priority compounds (247678791) caused a modest, but highly consistent reduction in released phosphate. The strong priority compound (247678835) could not be evaluated in this assay because it reacted with the malachite green dye, giving an apparent reaction product in the absence of enzyme/substrate.

### Human glioma tumor models in mice

Our ultimate goal is to identify compounds that have therapeutic potential for treating glioblastoma. To achieve this, we need to confirm that our compounds can affect tumor growth in vivo. We chose one pan-inhibitor (247678835) to test in a human glioma xenograft flank tumor model in mice. Compound 247678835 was the strongest inhibitor identified in the initial screen of LN229 migration. It was effective in every assay and was the strongest pan-inhibitor to affect PTPμ-dependent aggregation (Fig 7). It was also shown to be effective at inhibiting LN229 sphere growth down to 50 μM (S6 Fig), making it our primary candidate for in vivo testing.

Human LN229 glioma cells were subcutaneously injected into the flanks of twelve athymic nude mice (n = 6 per treatment group). Once tumors were established (12-days post cell injection), DMSO or 247678835 was injected into the center of each tumor once a week for three weeks, and tumor volumes were calculated from caliper measurements. Individual tumor volumes were normalized to their starting volumes and the data is displayed as % growth. Mice were sacrificed 4 weeks after the first treatment, and tumors were harvested, fixed, sectioned, and stained with H&E.

**Fig 9:**
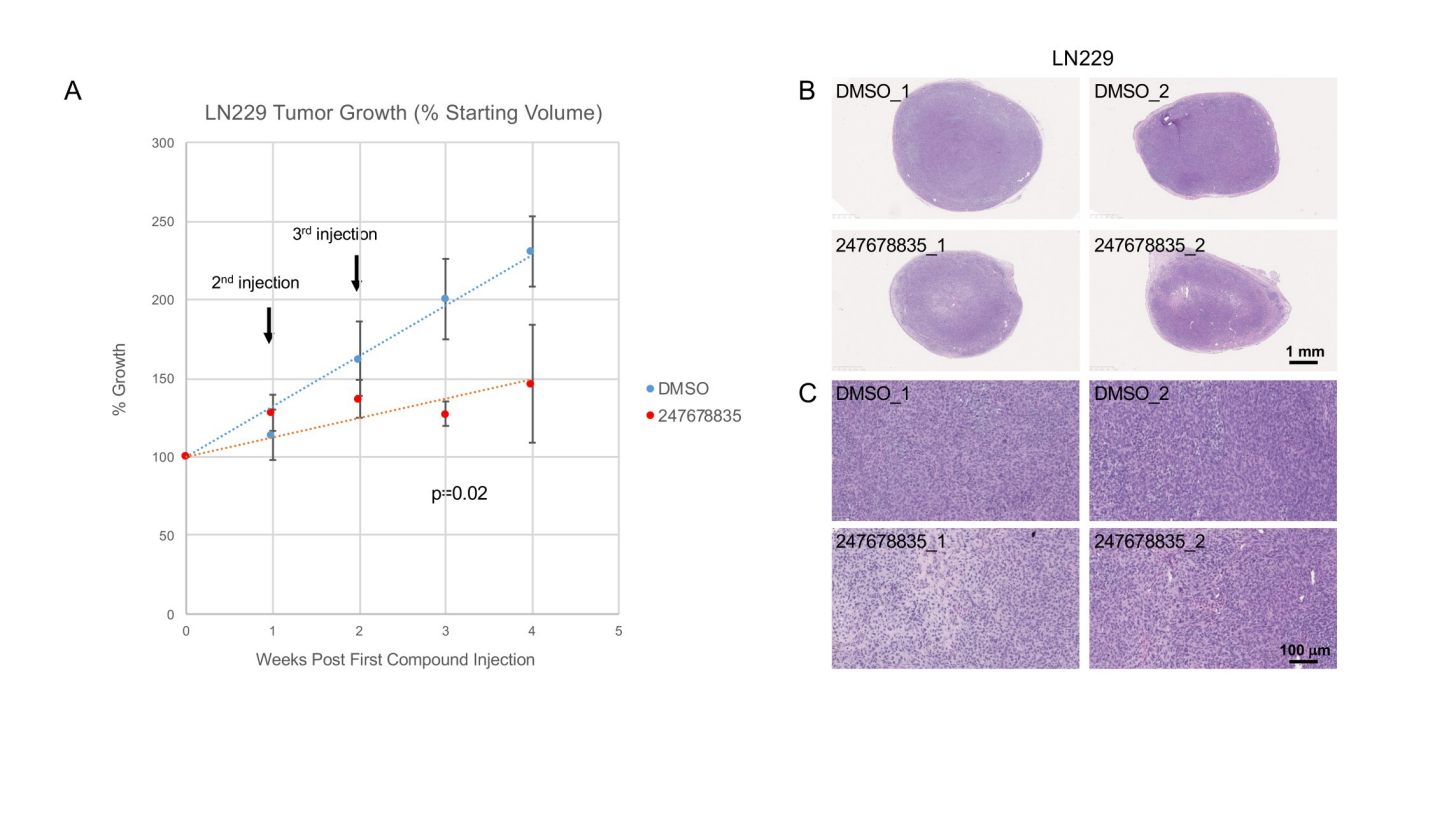
Human glioma tumor model in mice. LN229-flank tumors (n = 6 per treatment group) established in nude mice were injected with vehicle or compound once a week for three weeks. A. Tumor sizes were measured once a week for four weeks and normalized to their starting sizes. Data is presented as average percent growth ± s.e.m. Between-group comparisons were made using Student’s t-test. Differences were considered significant at p<0.05. 247678835 slowed tumor growth, producing a statistically significant growth reduction by 3 weeks post-first injection; however, growth seemed to rebound once treatment was stopped, and the slowed growth rate was no longer statistically appreciable at 4 weeks post-injection. Representative images of H&E-stained sections from tumors (two per treatment group) harvested at 4-weeks are shown. Tumors treated with 247678835 appeared smaller and less cellular based on the density of nuclei.

Treatment with 247678835 slowed tumor growth (Fig 9A). At 3 weeks post first injection, DMSO-treated tumors had doubled in size, but those treated with 247678835 had only increased slightly in size (1.3x). However, by four weeks post first injection, the tumors treated with 247678835 appeared to rebound, with some resuming growth after the treatment had been discontinued (note the increase in normalized size and increased error bars). Regardless, 247678835 tumors harvested at 4 weeks generally appeared less ‘cellular’ than DMSO-treated controls based on the density of nuclei in H&E stained sections (Fig 9B and 9C). In fact, 247678835 induced LN229-cell death in vitro (S8 Fig).

## Discussion

Through AI-based computational and functional screens we identified three high priority wedge-targeting compounds that inhibit PTPμ-dependent adhesion, glioma cell migration, and glioma sphere formation and growth with the results summarized in Fig 2. One of these compounds (247678791) was also shown to modestly inhibit the phosphatase activity of a PTPμ-intracellular construct, demonstrating a direct effect on PTPμ. Unfortunately, the strongest priority compound (247678835) could not be evaluated in our in vitro phosphatase assay system because it interacted with the dye used to measure the release of free phosphate. This compound was, however, shown to inhibit flank tumor growth in vivo, a necessary first step towards identifying compounds with therapeutic potential. Compound 247678835 was found to affect the survival of LN229 cells (which express PTPμ fragments) but not parental Sf9 cells (which lack PTPμ). We hypothesize that this compound might inhibit PTPμ fragment-dependent survival or migration signals.

We identified three additional interesting categories of compounds in this screen. We identified one compound (247685429) which was able to inhibit PTPμ-dependent adhesion but did not affect glioma cells. It is possible this compound can affect full-length PTPμ, perhaps by interfering with binding partners at the membrane, but not PTPμ fragments. Conversely, we identified four compounds (247677616, 247679515, 247679045 and 247682189) that were highly penetrant inhibitors in glioma cell assays but did not affect PTPμ-dependent adhesion. Compounds in this category could affect PTPμ fragments but not impinge on the activity of the full-length protein at the membrane perhaps by affecting binding partners. Regardless of mechanism, the inhibitory effects of these compounds on glioma cells means they are of therapeutic interest. Finally, we identified one activator of PTPμ-dependent adhesion that was weakly stimulatory in U87 and LN229 scratch assays and Gli36 sphere formation and growth assays (247685114). This was surprising, considering that restoring full-length PTPμ (and presumably PTPμ-dependent adhesion) to LN229 cells was shown to inhibit their motility [46]. However, a slight stabilization of PTPμ may stimulate contact-dependent chain cell migration. Alternatively, the compound that facilitates PTPμ-dependent adhesion in Sf9-PTPμ cells could interact in a stimulatory way with fragments in glioma cells.

The complexity of cell-based assays (which in this case involved several cell lines with differing ratios of PTPμ and full-length protein) makes it challenging to interpret the different behaviors of our compounds. However, we chose these assays over biochemical screening strategies for the following reasons: 1) Although an isolated wedge domain has been shown to mediate self-association in in vitro binding assays [24], it is unknown whether this happens in the context of either the full-length protein or its intracellular fragments; 2) The function of the wedge domain has been best characterized in cell-based assays, including the LN229 scratch assay [46], which was the basis of our primary screen; 3) Biochemical screening efforts to identify phosphatase inhibitors have limitations because compounds demonstrated to be effective in vitro often fail in vivo, in part due to membrane-permeability issues; 4) isolated assay systems cannot recapitulate all possible binding interactions necessary to reveal wedge-dependent effects. In fact, we had no *a priori* expectation that our screen would reveal compounds able to directly inhibit phosphatase activity or affect PTPμ-dependent adhesion. Constructs lacking the wedge domain induce aggregation [15, 19], and, although the juxtamembrane region was found to be required for PTPμ enzymatic activity [32], this was not precisely mapped to the wedge domain.

We can only hypothesize how the compounds identified in this screen might affect PTPμ’s functions. In regards to PTPμ-dependent adhesion, dimerization/oligomerization in the plane of the membrane is involved in stabilization of adhesion [15], and if the wedge domain participates in intermolecular interactions between PTPμ molecules, interfering with this might inhibit PTPμ’s adhesive activity. Consistent with this, wedge peptides have been shown to self-associate [24], suggesting they could mediate trans interactions between PTPμ molecules. Considering enzymatic activity, the predicted compound binding pocket is approximately 20 Å away from the catalytic domain. This distance is similar to that of allosteric binding pockets identified in other phosphatase family members that are predicted to act by altering the flexibility of structures surrounding the active site that are necessary for catalysis [4].

In conclusion, we have identified small molecules predicted to interact with a pocket adjacent to the wedge domain of PTPμ that inhibit glioma cell migration, growth in 3D culture, PTPμ-dependent adhesion, and for one compound (247678791), phosphatase activity. Future directions will focus on direct binding assays to confirm whether/how these compounds interact with PTPμ and how this might affect downstream pathways important for glioma cell motility, survival, and/or growth. Structure activity relationship studies are also needed to optimize lead compounds. Finally, although we have preliminary evidence that one compound (247678835) was able to affect tumor growth *in vivo*, this was done with direct injection into the tumor. Future work is needed to establish if this compound can be administered orally or systemically.

## Materials and methods

### Cell culture

Sf9 insect cells and the human glioma cell lines LN229 (LN-229) and U87 (U-87 MG) were obtained from ATCC. The Gli36 (Gli36δ5) [61] human glioma line was obtained from E. Chiocca and authenticated using IDEXX BioResearch (formerly RADIL: Research Animal Diagnostic Laboratory at the University of Missouri). Gli36 and U87 cells were cultured in DMEM (High Glucose DMEM, Gibco, Grand Island, NY) + 10%FBS (HyClone, South Logan, UT), and LN229s were cultured in DMEM + 5%FBS. All glioma cell lines were maintained at 37° C and 5% C0_2_. Sf9 cells were cultured in Grace’s Complete Medium (Gibco, Grand Island, NY) +10% FBS at 27° C.

### Scratch wound assays

Cells were seeded at a density of 2.7x10^4^ cells per well into the internal wells of Incuyte® Imagelock 96-well plates (Essen BioScience Inc., Ann Arbor, MI) and cultured overnight to form monolayers. Monolayers were wounded with an IncuCyte® 96-well Woundmaker Tool per the manufacturer’s instructions. The outer wells were filled with PBS to buffer edge effects, and then the wounded monolayers were cultured in 100 μl of fresh media with compounds or DMSO (2x replicates at 100 μM or 1%, respectively) at 37° C and 5% CO_2_. Images were captured every 4 hrs. using an Incucyte live cell imaging system equipped with the Scratch Wound Module. Scratch wound widths were calculated, per the manufacturer’s instructions, at T0 and at endpoint (typically T12 for LN229 and U87 cells and T8 for Gli36), which was taken as the last timepoint before wound closure. The cell migration distance was calculated from scratch widths [(T_0_Width-T_endpoint_Width)/2] and normalized to the average distance migrated by the DMSO controls. Values are presented as average percentages ± standard errors of the means (s.e.m.). The majority of compounds were screened with an n of two, but the n for priority hits ranges from 2–6.

### Glioma sphere assays

Cells were seeded at a density of 7500 cells per well into the internal wells of 96-well plates coated with 0.75% (wt/vol) PVA as previously described [59]. Compounds were added (2x replicates per treatment) at the indicated final concentrations, and control wells were treated with matching concentrations of DMSO. The external wells of the plates were filled with PBS to buffer against edge effects, and the cells were incubated at 37° C and 5% CO_2_ for 7 days. A Leica CTR6500 microscope fitted with an automated stage was used to capture brightfield images on day 1 and day 7, and sphere footprint areas were measured using Image J (v1.52a http://imagej.nih.gov/ij) as previously described [59]. To quantify the effects of the compounds on day 1, the footprint areas of the treated wells were normalized to the average area of the matched DMSO control wells. To quantify the effects of the compounds on sphere growth, the change in the sphere footprint areas was calculated (day1/day7*100) and then normalized to the average size change of the matched DMSO samples. All values are presented as average percentages ± s.e.m.

### Helix blue staining

LN229 cells, plated onto non-adherent surfaces as described above, and parental Sf9 cells (without PTPμ), seeded into 96-well flat bottom tissue culture plates, were treated for 24 h with the indicated compounds (100 μM). The cells and spheres were then treated with 5.5 μM Helix Blue (Biolegend, San Diego, CA) and imaged at 10x on a Leica CTR6500 fluorescence microscope.

### PTPμ-dependent aggregation assay

Sf9 cells were infected with baculovirus coding for human full-length PTPμ [22] and induced to aggregate following a modification of the procedure described in Brady-Kalnay et al. (1993) [18]. This is a new high throughput 48 well based aggregation assay for drug screening [55]. Briefly, 40 h after infection, cells (both floating and adherent) were gently triturated to separate clumps, and 1.14x10^4^ cells per well were seeded into 48-well culture plates pre-treated with 0.75% (wt/vol) PVA to prevent cells from adhering to the plastic [59]. Compounds were added (2x replicates per treatment) and bubbles removed by puffing air across the plate. Each well contained a final volume of 180 μl media with compounds (at 100 μM) or DMSO (at 1%). The plates were incubated at room temperature for 20 min then rotated at 120 rpm for 30 min to induce aggregation. To facilitate automated image analysis, aggregates and loose cells, which typically swirl to the center of the wells, were distributed by manually shaking the plate before imaging the entire surface area of each well by capturing a 4x4 grid of images using a Leica CTR6500 microscope with an automated stage and a 5x objective. Image J was used to quantify the number of aggregates per well with footprint areas > 4000 μm^2^.

run("Set Scale…", "distance = 0.274 known = 1 pixel = 1 unit = microns global");

run("Subtract Background…", "rolling = 1 light");

run("Invert");

run("Smooth");

run("Smooth");

setAutoThreshold("Default dark");

setOption("BlackBackground", true);

run("Convert to Mask");

run("Fill Holes");

run("Make Binary");

run("Analyze Particles…", "size = 4000–60000 show = Masks summarize");

The automated counts were manually corrected for errors arising from touching aggregates, piles of loose cells, or debris.

### In vitro phosphatase assay

A GST fusion protein containing the intracellular domain of PTPμ (B5: aa 765–1449) [62] (PTPμ_intra) was prepared as described [40]. Phosphatase reactions (50 μl total volume) were assembled on ice by mixing 0.4 μg GST-PTPμ_intra with phosphatase buffer (25 mM Hepes pH 7.4, 50 mM NaCl and 2 mM DTT) and compounds (100 μM) or DMSO (1%). Samples were incubated 10 min on ice and then reactions started by adding the peptide substrate (DADEY(PO3)LIPQQG, R&D Systems, Minneapolis, MN) to a final concentration of 60 μM and transferring the tubes to a circulating water bath at 30° C. Reactions were stopped at 10 min by addition of malachite green dye stock (Malachite Green Phosphatase Assay Kit, Sigma-Aldrich, St. Louis, MO) prepared per the manufacturer’s instructions. Colorimetric product was allowed to develop for 15 min at room temperature, and the absorbance of the samples and a standard curve of free phosphate (assembled per the manufacturer’s instructions) were read at 600 nm on a Synergy HT Microplate Reader (BioTek Instruments Inc., Winooski, Vermont). The amount of released phosphate was calculated, normalized to that released by the DMSO control sample, and expressed as a percent. The data presented is the average of 3–6 independent experiments.

### Tumor growth assay

Glioma-cell tumor xenografts were prepared as previously described [63]. Briefly, LN229 cells (2x10^6^ per injection) mixed with Matrigel (Corning, Corning Inc., Corning, NY, USA) were subcutaneously injected into the flank of athymic nude (FoxN1^nu^/Foxn1^nu^) female mice bred by the Case Western Reserve University Athymic Animal Core Facility or obtained from The Jackson Laboratory (Bar Harbor, ME). Experiments were approved by our IACUC committee. Twelve days post tumor-cell-injection, 247678835 or DMSO was diluted into PBS to give final concentrations of 2 mM or 20%, respectively, and 25 μl was injected into the center of each flank tumor. Additional compound or DMSO injections were given 7 and 14 days after the first injection, and tumor volumes [(length × width^2^)/2] were recorded once a week for 4 weeks. At four weeks, mice were sacrificed, and tumors were isolated, fixed in 10% buffered formalin, and prepared for paraffin sectioning. Five micron-thick sections were cut, stained with H&E, and images captured with a Hamamatsu Nanozoomer S60 Slide Scanner (Hamamatsu Photonics, K.K., Bridgewater, NJ, USA).

## Supporting information

S1 Fig Representative endpoint images of LN229 scratch wounds treated with DMSO or selected priority compounds. A-F. Endpoint images of samples treated with DMSO or the indicated inhibitors. G. Endpoint images of a sample treated with a weak activator. The distance moved relative to controls for each example is indicated.
(TIF)

S2 Fig U87 scratch wound assays. A. Histogram showing the effects of all soluble wedge pocket-targeting compounds on U87 scratch wound closure. Cell movement into the scratches was quantified from scratch wound widths at the start and end of the assay and normalized to the average movement of cells in the unblinded DMSO control samples. Data is presented as average percentages ± s.e.m., and compound bar codes are shown on the x-axis. Most compounds were screened with an n of 2–4. Representative images of scratch wounds treated with DMSO (A) or two priority inhibitors (C and D) are shown.
(TIF)

S3 Fig Representative endpoint images of U87 scratch wounds treated with DMSO or selected priority compounds. A-F. Endpoint images of samples treated with DMSO or the indicated inhibitors. G. Endpoint images of a sample treated with a weak activator. The distance moved relative to controls for each example is indicated.
(TIF)

S4 Fig Representative examples of compounds (100 μM) that exhibited insolubility in scratch and sphere assays. (TIF)

S5 Fig Representative images of Gli36 scratch wounds treated with the indicated priority compounds. A-F. Endpoint images of samples treated with DMSO or the indicated inhibitors. G. Endpoint images of a sample treated with a weak activator. The distance moved relative to controls for each example is indicated.
(TIF)

S6 Fig Titration of selected compounds on LN229 sphere formation and growth. LN229 cells were plated onto non-adherent surfaces and treated with the indicated compounds at 100, 50, and 25 μM. A. On day 1, sphere footprint areas were determined and normalized to the average footprint area of the unblinded vehicle-treated controls. On day 1, a larger footprint area indicates inhibition of aggregation. B. On day 7, the changes in sphere footprint areas were calculated and normalized to the average size change of the unblinded vehicle-treated controls. On day 7, a smaller value indicates reduced growth. Growth could not be calculated for samples that fell apart on day 1 or during the assay, and this is indicated as ‘0’ growth. Data is presented as percentages ± s.e.m. The initial test at 100 μM and the follow-up at that dose with titration is shown. Each bar is the average of 2 replicates. Representative day 1 (C) and day 7 (D) images of samples treated with two priority inhibitors are shown. Relative day 1 sphere footprint areas and day 7 growth for each example are indicated.
(TIF)

S7 Fig Representative images of LN229 and Gli36 spheres cultured in the presence of DMSO or the indicated priority compounds (100 μM). The relative day 1 footprint area and day 7 size change for each compound are indicated.
(TIF)

S8 Fig Testing the effects of prioritized inhibitors on cell survival. LN229 cells were plated onto non-adherent surfaces and cultured in the presence of the indicated compounds (100 μM). On day 1, spheres were stained with Helix Blue to detect dying cells. Parental Sf9 cells (which lack PTPμ) plated onto tissue culture plastic were also grown in the presence of the indicated compounds and, on day 1, stained with Helix Blue. Three compounds appeared to cause a qualitative increase in staining in LN229 spheres. No compound was toxic to Sf9 cells. There is variability in the level of Helix Blue staining exhibited by LN229 control spheres, so two untreated examples are shown.
(TIF)
