# Supplementary figures and images for: Small molecule antagonists of PTPmu identified by artificial intelligence-based computational screening block glioma cell migration and growth
Source: PLoS One. 2023 Jul 26;18(7):e0288980. doi: 10.1371/journal.pone.0288980 (PMC10370706; doi:10.1371/journal.pone.0288980)

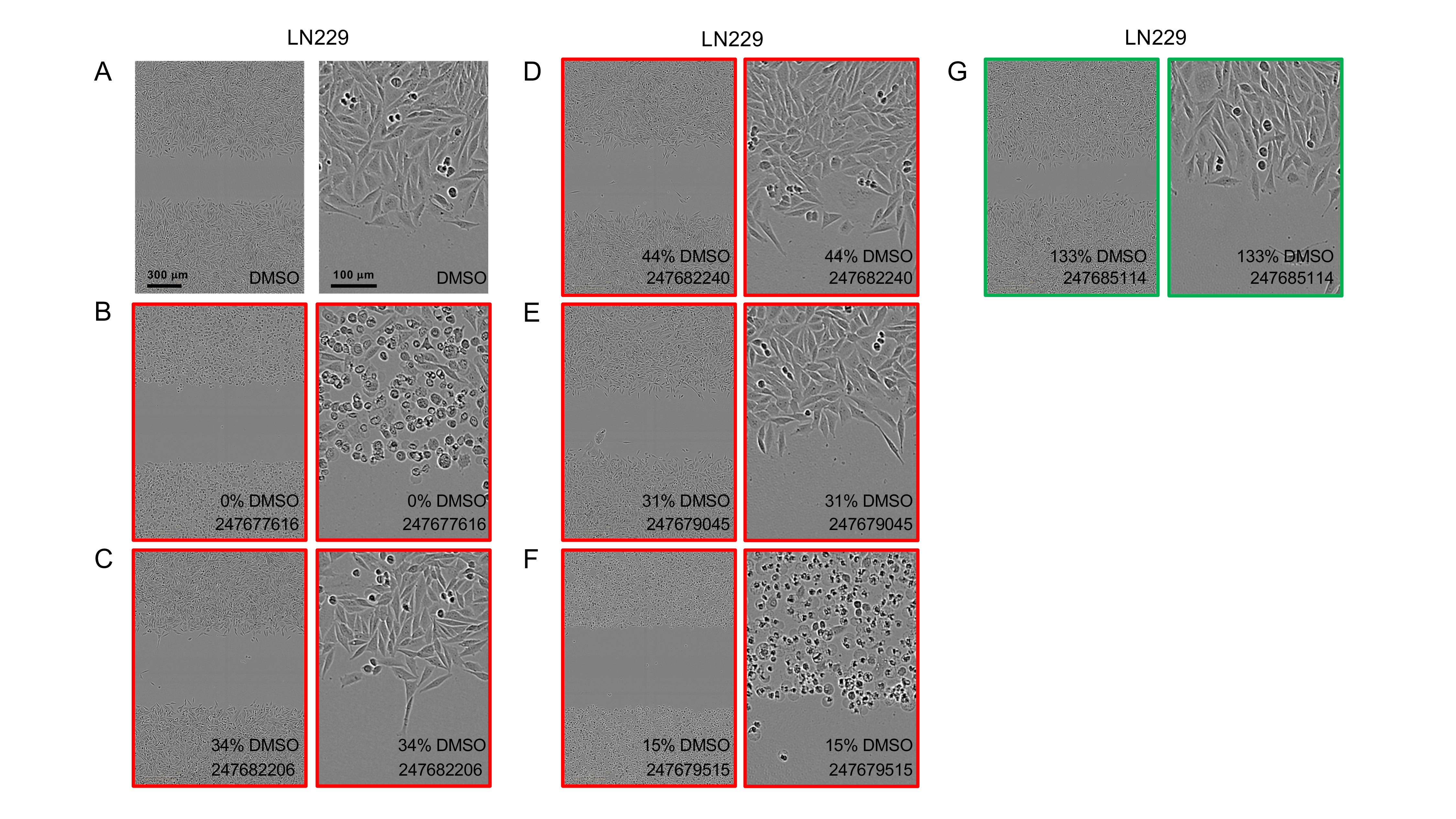

Supplement: S1 Fig — A-F. Endpoint images of samples treated with DMSO or the indicated inhibitors. G. Endpoint images of a sample treated with a weak activator. The distance moved relative to controls for each example is indicated. (TIF) [file pone.0288980.s001.tif]

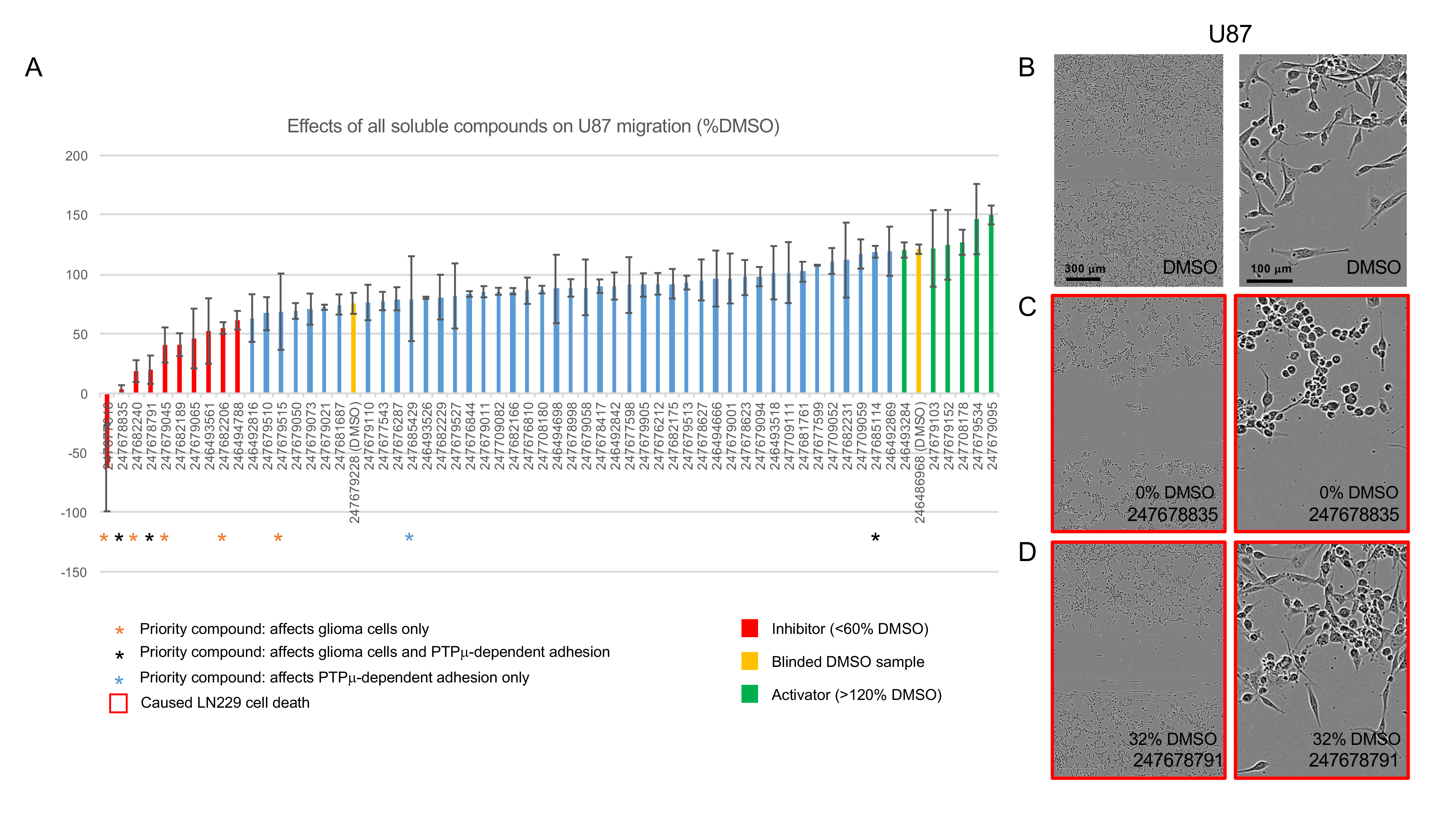

Supplement: S2 Fig — A. Histogram showing the effects of all soluble wedge pocket-targeting compounds on U87 scratch wound closure. Cell movement into the scratches was quantified from scratch wound widths at the start and end of the assay and normalized to the average movement of cells in the unblinded DMSO control samples. Data is presented as average percentages ± s.e.m., and compound bar codes are shown on the x-axis. Most compounds were screened with an n of 2–4. Representative images of scratch wounds treated with DMSO (A) or two priority inhibitors (C and D) are shown. (TIF) [file pone.0288980.s002.tif]

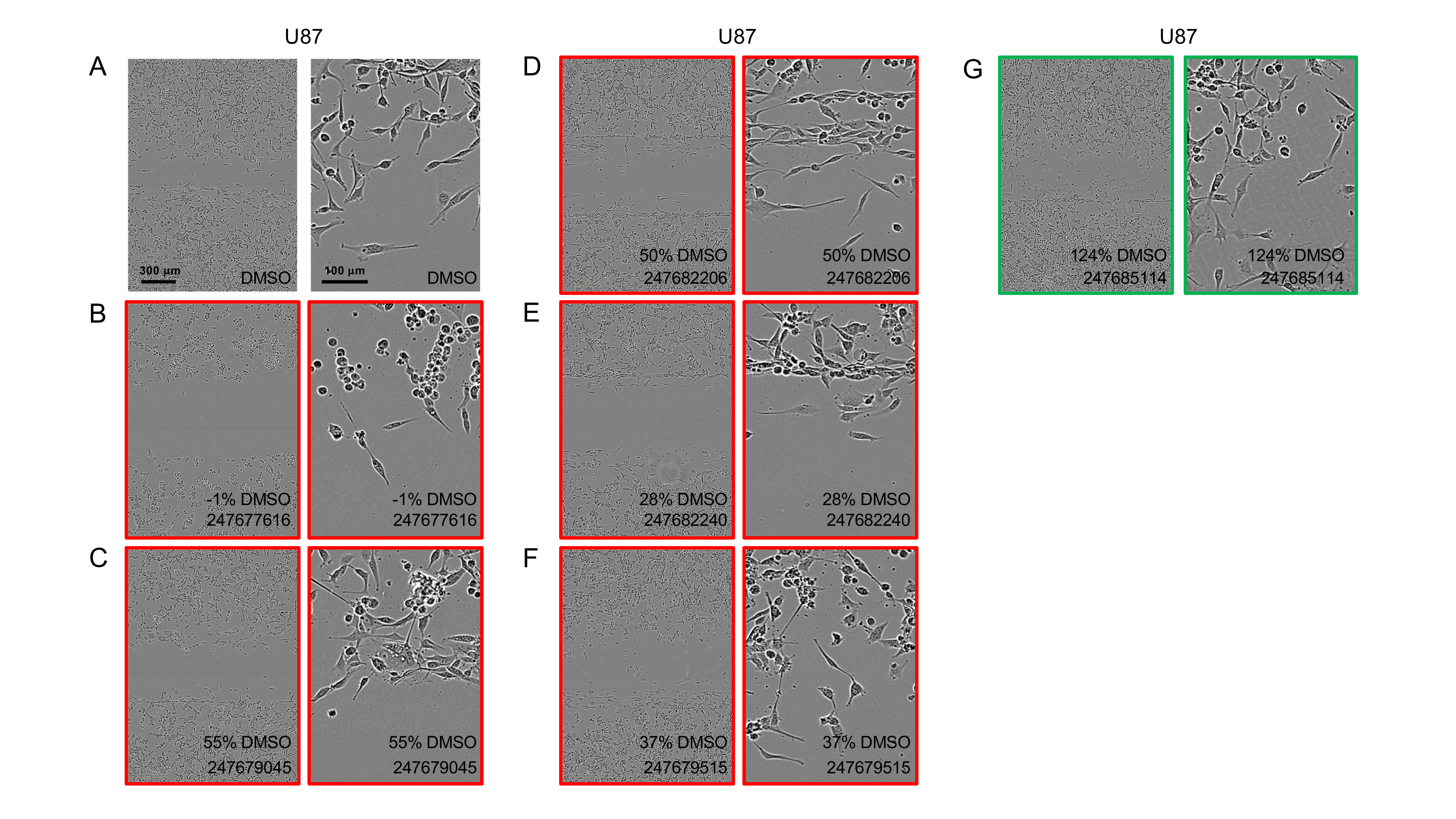

Supplement: S3 Fig — A-F. Endpoint images of samples treated with DMSO or the indicated inhibitors. G. Endpoint images of a sample treated with a weak activator. The distance moved relative to controls for each example is indicated. (TIF) [file pone.0288980.s003.tif]

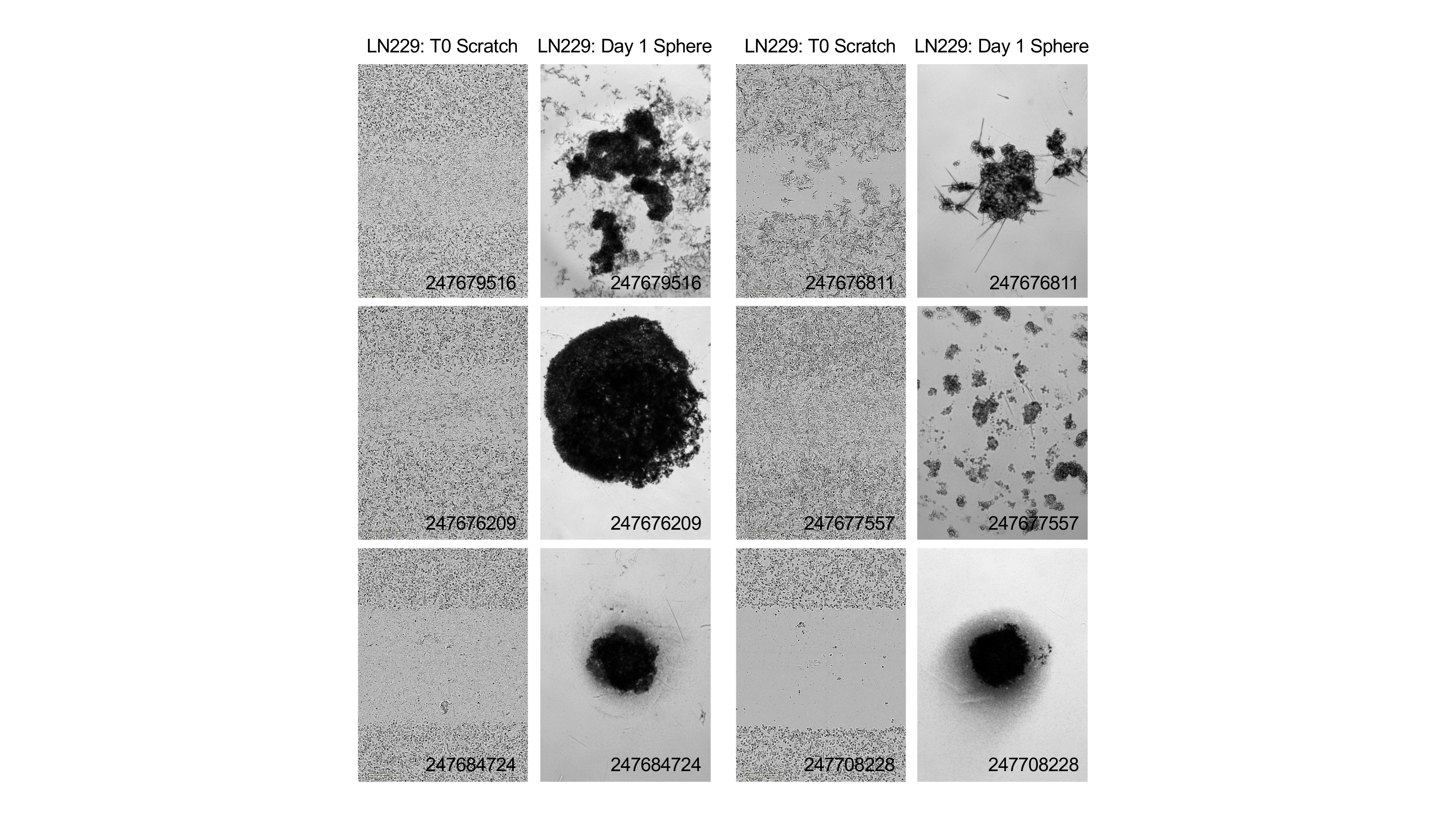

Supplement: S4 Fig — (TIF) [file pone.0288980.s004.tif]

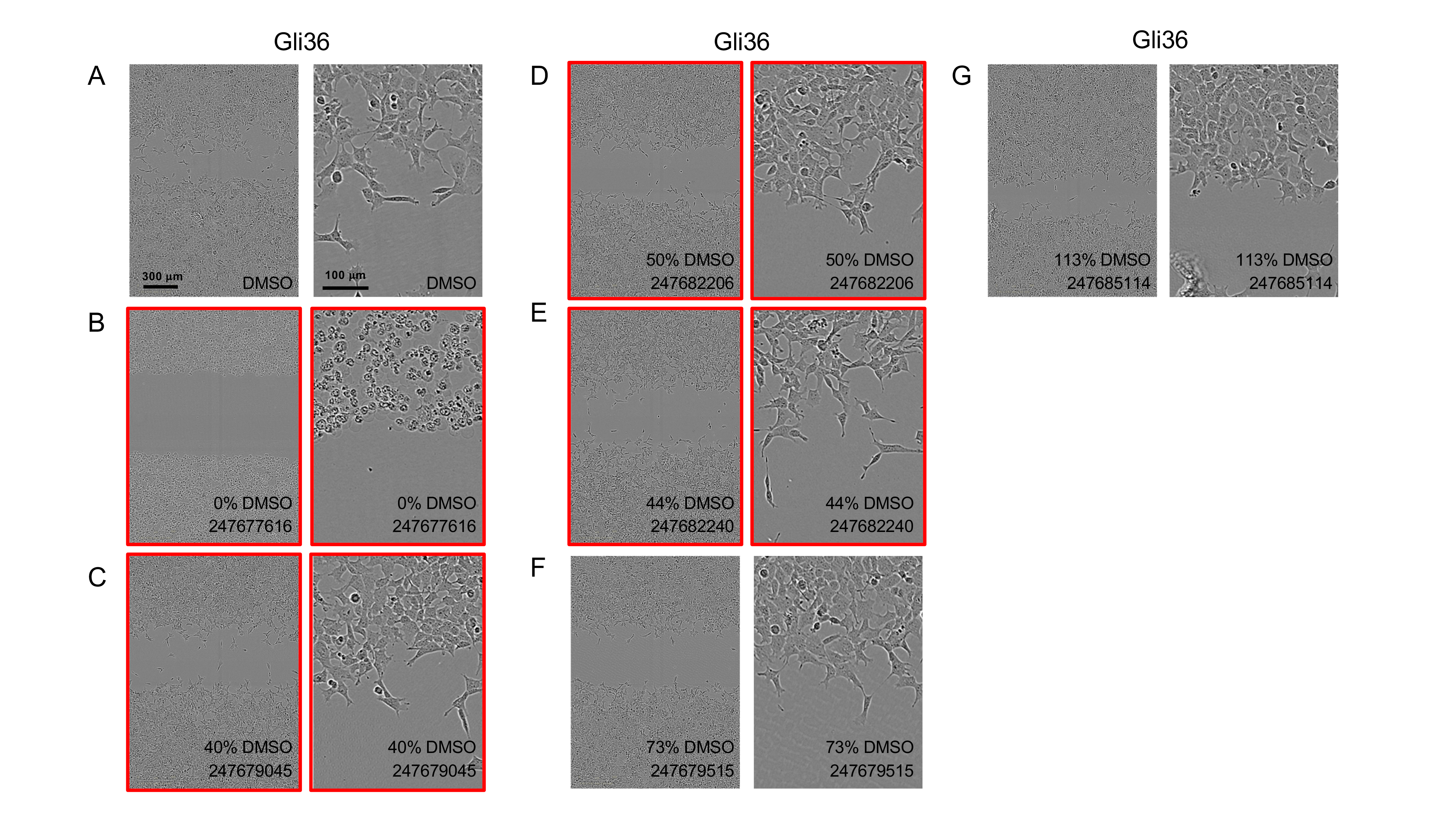

Supplement: S5 Fig — A-F. Endpoint images of samples treated with DMSO or the indicated inhibitors. G. Endpoint images of a sample treated with a weak activator. The distance moved relative to controls for each example is indicated. (TIF) [file pone.0288980.s005.tif]

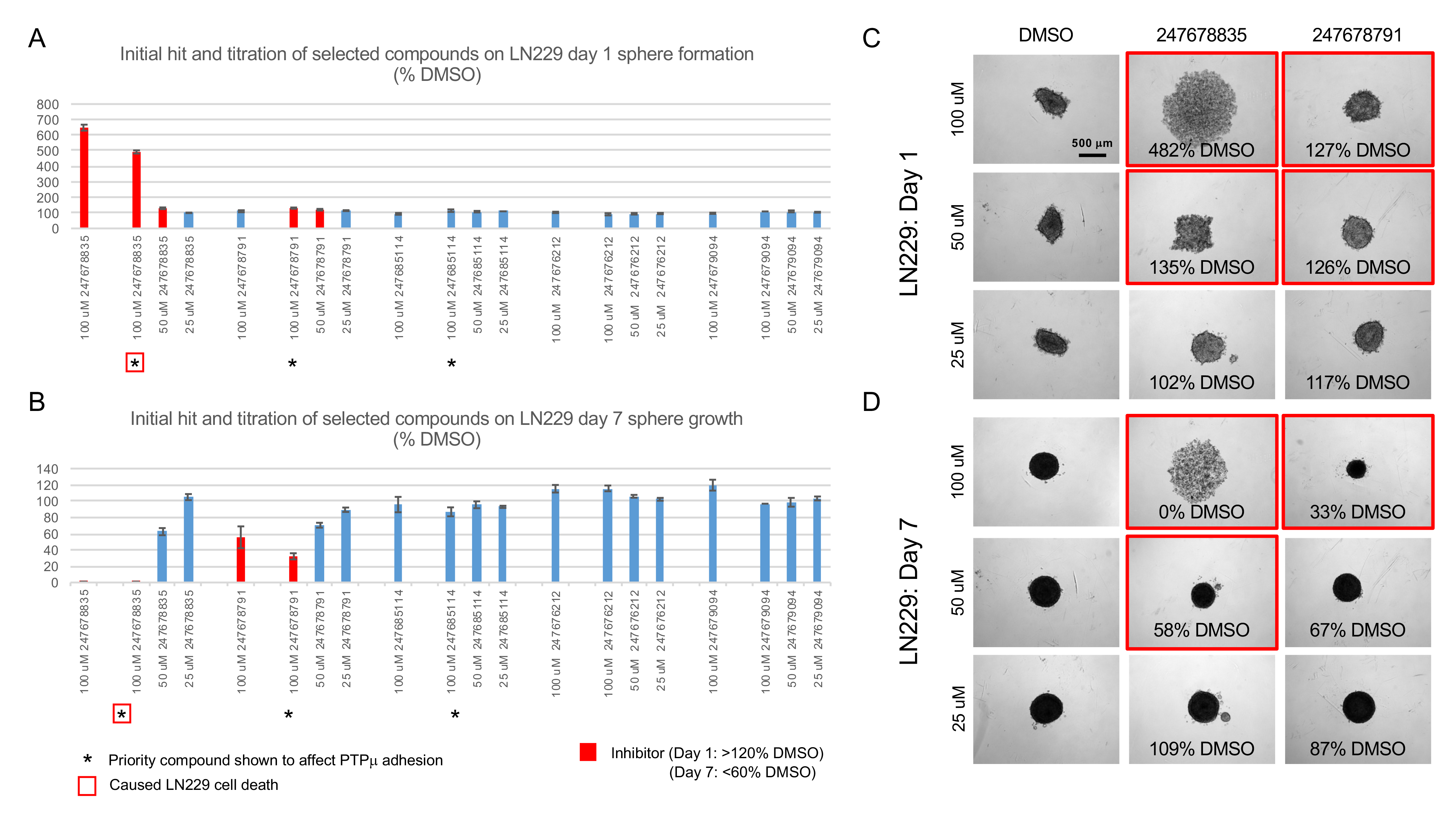

Supplement: S6 Fig — LN229 cells were plated onto non-adherent surfaces and treated with the indicated compounds at 100, 50, and 25 μM. A. On day 1, sphere footprint areas were determined and normalized to the average footprint area of the unblinded vehicle-treated controls. On day 1, a larger footprint area indicates inhibition of aggregation. B. On day 7, the changes in sphere footprint areas were calculated and normalized to the average size change of the unblinded vehicle-treated controls. On day 7, a smaller value indicates reduced growth. Growth could not be calculated for samples that fell apart on day 1 or during the assay, and this is indicated as ‘0’ growth. Data is presented as percentages ± s.e.m. The initial test at 100 μM and the follow-up at that dose with titration is shown. Each bar is the average of 2 replicates. Representative day 1 (C) and day 7 (D) images of samples treated with two priority inhibitors are shown. Relative day 1 sphere footprint areas and day 7 growth for each example are indicated. (TIF) [file pone.0288980.s006.tif]

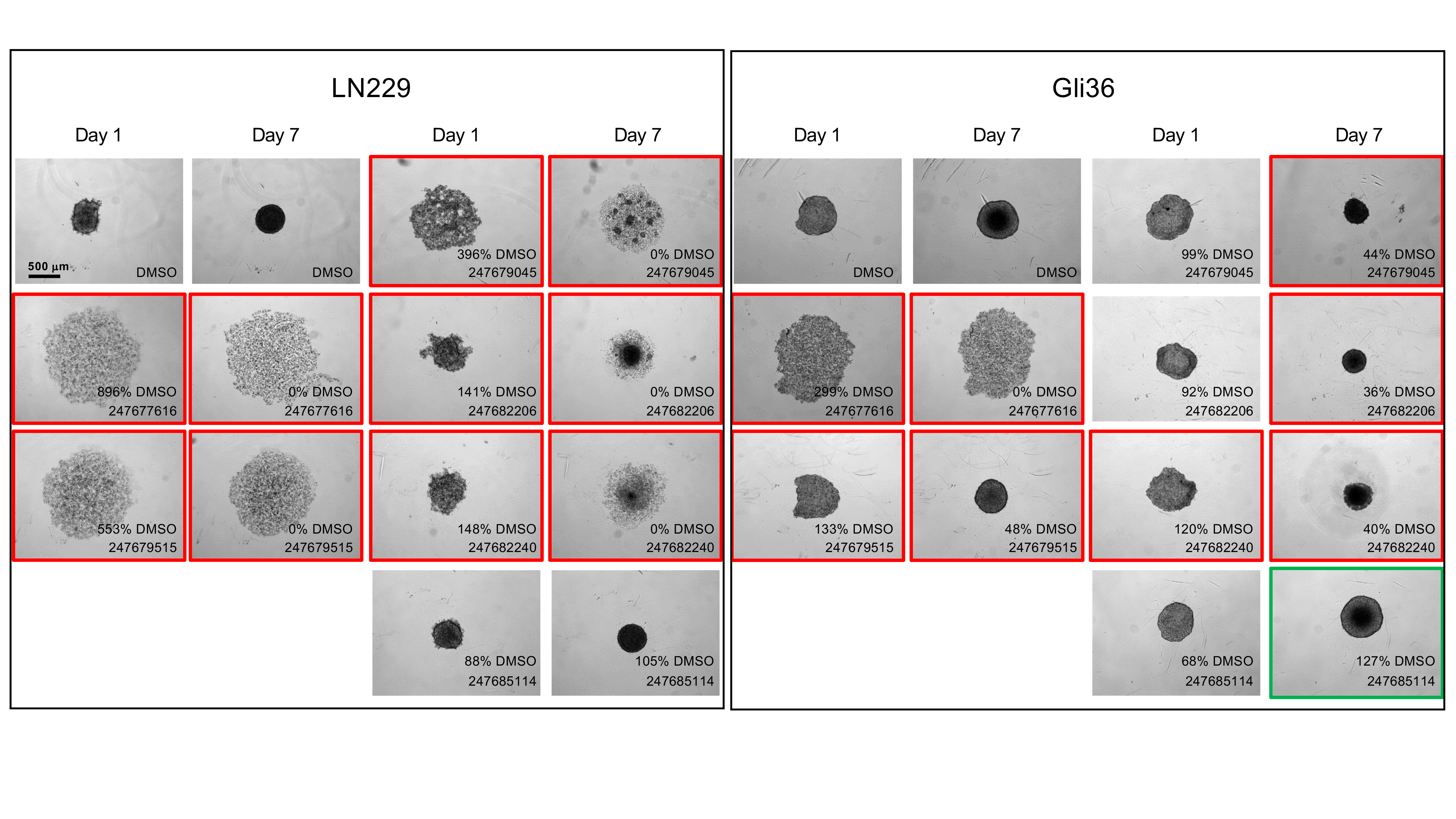

Supplement: S7 Fig — The relative day 1 footprint area and day 7 size change for each compound are indicated. (TIF) [file pone.0288980.s007.tif]

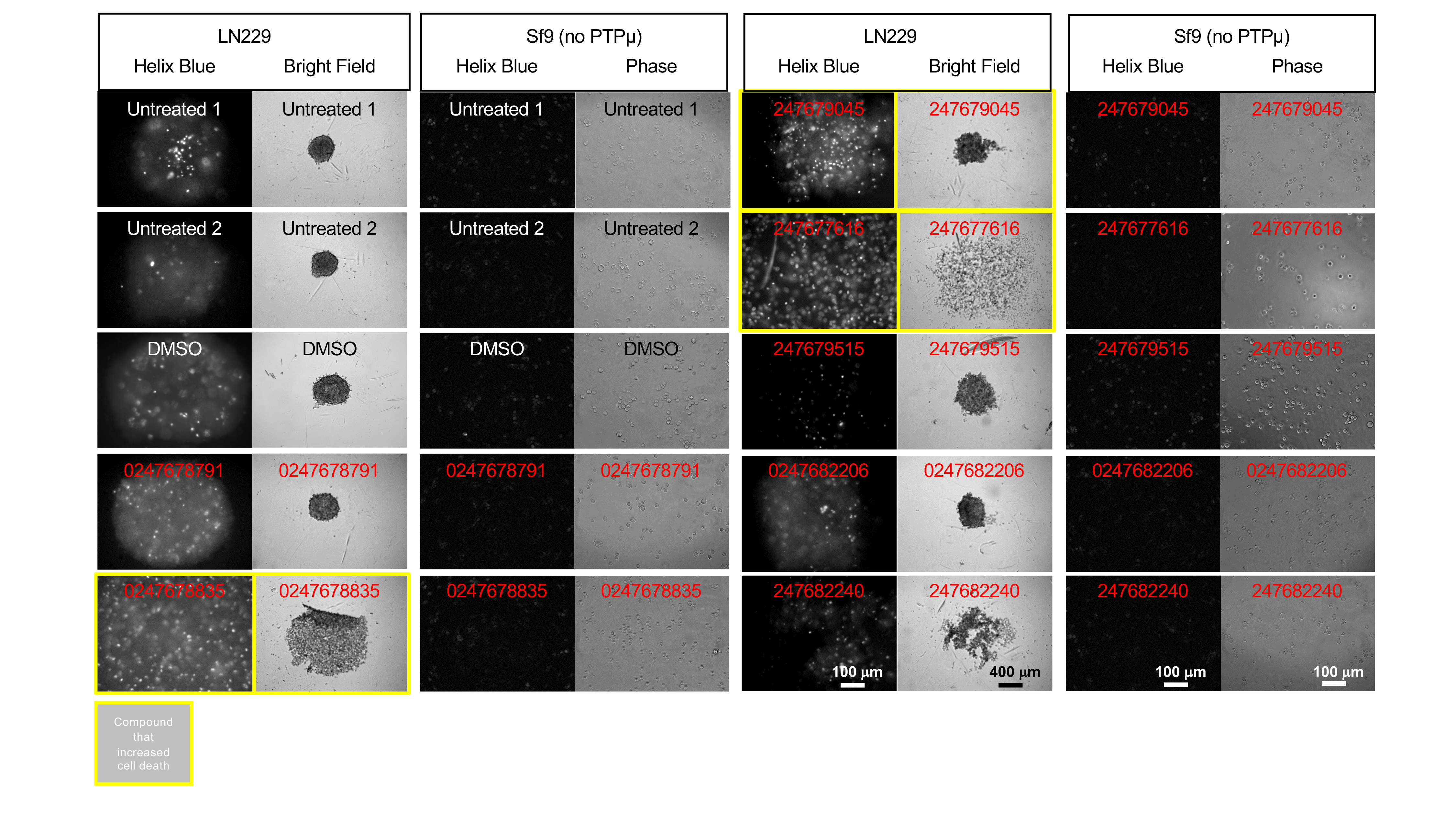

Supplement: S8 Fig — LN229 cells were plated onto non-adherent surfaces and cultured in the presence of the indicated compounds (100 μM). On day 1, spheres were stained with Helix Blue to detect dying cells. Parental Sf9 cells (which lack PTPμ) plated onto tissue culture plastic were also grown in the presence of the indicated compounds and, on day 1, stained with Helix Blue. Three compounds appeared to cause a qualitative increase in staining in LN229 spheres. No compound was toxic to Sf9 cells. There is variability in the level of Helix Blue staining exhibited by LN229 control spheres, so two untreated examples are shown. (TIF) [file pone.0288980.s008.tif]
